# Improved variants of SrtA for site-specific conjugation on antibodies and proteins with high efficiency

**DOI:** 10.1038/srep31899

**Published:** 2016-08-18

**Authors:** Long Chen, Justin Cohen, Xiaoda Song, Aishan Zhao, Zi Ye, Christine J. Feulner, Patrick Doonan, Will Somers, Laura Lin, Peng R. Chen

**Affiliations:** 1 Beijing National Laboratory for Molecular Sciences, College of Chemistry and Molecular Engineering, Synthetic and Functional Biomolecules Center, Peking University, Beijing 100871, China; 2 Department of Global Biotherapeutics Technologies, Pfizer Inc., Cambridge, MA 02140, USA; 3 School of Life Sciences, Nanjing University, China; 4 Peking-Tsinghua Center for Life Sciences, Beijing, China

## Abstract

Sortase mediated ligation is a highly specific platform for conjugation that relies on the specificity of the transpeptidase Sortase A (SrtA) for short peptide sequences (LPXTG and GGG). SrtA retains its specificity while accepting a wide range of potential substrates, but its broad use is limited by the wild-type enzyme’s poor kinetics, which require large amounts of SrtA and extended reaction times for efficient conjugation. Prior explorations have aimed to improve the kinetics of SrtA with limited success. Herein we describe the discovery of further improved SrtA variants with increased efficiency for the conjugation reaction, and demonstrate their robustness in labelling proteins and antibodies in a site-specific manner. Our variants require significantly lower amounts of enzyme than WT SrtA and can be used to attach small molecules to the N or C-terminus of the heavy or light chain in antibodies with excellent yields. These improved variants can also be used for highly efficient site-specific PEGylation.

*S. aureus* Sortase A (SrtA, SA-SrtA, SrtA_staph_) is a transpeptidase that has been widely adopted for site-specific protein modification and engineering. The reaction catalyzed by SrtA results in the formation of a new amide bond between a C-terminal sorting motif LPXTG (X equals any amino acid) and an N-terminal oligoglycine. The conjugation reaction proceeds by first cleaving the peptide bond between the threonine and glycine residues within the sorting motif^1^,^2^,^3^, and is outlined in Fig. 1A.

By appending one of the sorting motifs to a target protein, one can use sortase to site-specifically modify the protein for a variety of applications. Examples of applications of sortase mediated ligation include protein cyclization, solid-support immobilization, PEGylation, and fluorescent tagging^3^,^4^,^5^,^6^. Labeling with SrtA has been proven to be successful at both the protein N and C terminus^3^. In addition, a non-canonical protein internal lysine modification has recently been found to be feasible using SrtA^7^.

One particularly exciting application where the site-specific nature of sortase mediated ligations could be applied is for the generation of antibody drug conjugates (ADCs). ADCs combine the high potency of small molecule drugs with the excellent specificity of antibodies towards cancer cells. The components of ADCs are relatively simple: an antibody to target a cancer cell surface antigen, a cytotoxic drug to kill the cancer cells, and a linker to join the drug and antibody^8^,^9^.

For the conventional production of ADCs, cytotoxic agents were conjugated to antibodies through nonspecific alkylation of cysteine (as for Adcetris)^10^ or acylation of lysine (as for Mylotarg and Kadcyla)^11^,^12^. However, these conjugations produce heterogeneous products with drug-to-antibody ratios (DAR) ranging from 0 to 8 as there are many accessible lysine and cysteine residues on the antibody surface^13^. In addition to product heterogeneity, the high DAR species in conventional ADCs have potential liabilities including faster clearance and unwanted toxicity through non-specific uptake and/or premature loss of the payload^14^. More recently site-specific conjugation strategies have been used to generate ADCs that are homogenous and have a defined DAR with the potential to greatly affect the properties of the final conjugate^15^,^16^,^17^,^18^,^19^,^20^,^21^,^22^,^23^,^24^,^25^,^26^,^27^. The inherent site-specificity of enzyme based conjugation approaches makes them an attractive method for these conjugations^28^,^29^,^30^,^31^,^32^,^33^.

Despite the strengths of SrtA as a conjugation platform, namely the high specificity for the LPXTG motif and the wide range of oligo-glycine substrates which the enzyme can accept, one of the main limitations of this technology is the relative inefficiency of the SrtA enzyme itself. This results in high amounts of SrtA or long reaction times being required in order to compensate for the inefficient kinetics of the enzyme. A yeast display based evolution process has illustrated the potential to increase the efficiency of Sortase^34^, and its use to produce ADCs has been recently reported^32^. However, the conjugation was incomplete and the conjugation sites restricted to the C-terminus of the antibody. In addition, conjugation to the C-terminus of the light chain was found to be particularly challenging and required higher SrtA concentrations and necessitated further engineering of a flexible amino acid spacer (e.g. (Gly_4_Ser)_n_)^35^ between the C-terminus and the sortase tag to improve conjugation efficiency.

Herein, we aimed to further evolve the Sortase A enzyme to improve and broaden its conjugation efficiency on both proteins and antibodies, and expand its general usefulness as a site-specific conjugation platform. We also sought to demonstrate the robustness of these improved variants in labelling proteins and to explore their use in labelling multiple sites in an antibody for potential ADC applications.

## Results

### Design of a FRET-based platform to measure Sortase A activity

**Figure 1:**
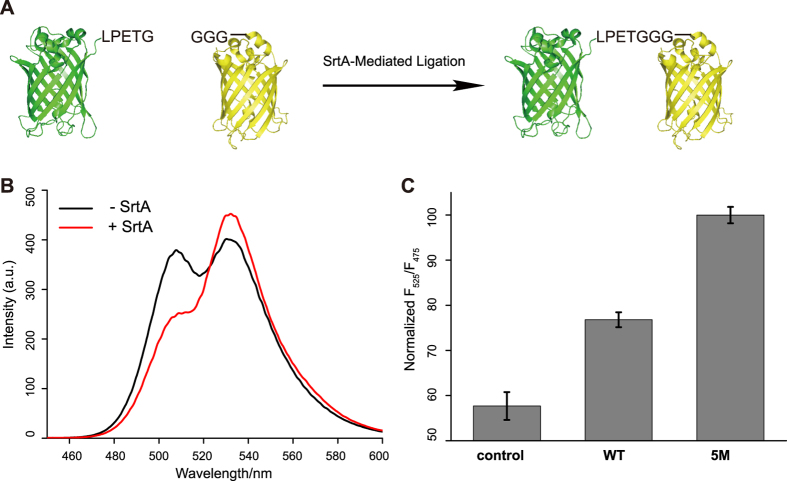
A FRET-based platform is able to report SrtA activity. **(A)** Design of the (fluorescence resonance energy transfer) FRET-based platform. Sorting motif LPETG was fused to the C-terminus of EGFP (green) and oligoglycine (GGG) was appended to the N-terminus of cpVenus (yellow). **(B)** Validation of the FRET-based platform for reporting SrtA activity. Enhanced FRET was observed in the presence of SrtA (red line) compared with absence of SrtA (black line). **(C)** Further validation of the platform with fluorescence ratio of 525 nm and 475 nm as readout. The 5M mutant was used as positive control and its fluorescence ratio was normalized to 100. Error bars represent ±s.d. from the mean of three parallel experiments.

*S. aureus* Sortase A is a bond-forming enzyme which can catalyze amide bond formation between both protein/protein and protein/small molecule. Inspired by previous research, we sought to design a highly sensitive, fluorescence resonance energy transfer (FRET) -based screening platform for high throughput screening of high efficiency SrtA variants. A FRET pair of fluorescent proteins (EGFP and cpVenus) was utilized, with the Sortase A sorting motif LPETG appended to the carboxyl terminus of EGFP and the nucleophilic attacking oligoglycine (e.g. GGG) fused to the amino terminus of cpVenus, respectively (Fig. 1A). Upon SrtA-mediated ligation, the FRET signal between donor protein EGFP and acceptor protein cpVenus will be enhanced (as shown in Fig. 1B) validating the suitability of the screening platform for assessing the enzymatic activity of SrtA. For our high throughput screening process, the fluorescence ratio at 525 nm and 475 nm was measured as the enzymatic activity readout, and the previously reported pentamutant (P94R/D160N/D165A/K190E/K196T, referred as 5M hereafter)^34^ was used as a positive control (Fig. 1C).

### Directed evolution of *S. aureus* Sortase A with FRET-based screening platform

We employed two complementary approaches to generate SrtA mutagenesis libraries for directed evolution. First, we used error-prone PCR to construct random mutation library on the WT-StrA template (Supplementary Figure 1). A random library of ~10^5^ transformants was generated. Second, based on the structural information and the mechanism of SrtA-mediated ligation, we performed saturated mutagenesis at a set of rationally selected sites either on WT SrtA or on 5M gene (Supplementary Figure 2). All of the screening processes were conducted in a 96-well plate format (detailed in Supplementary Figure 3).

**Figure 2:**
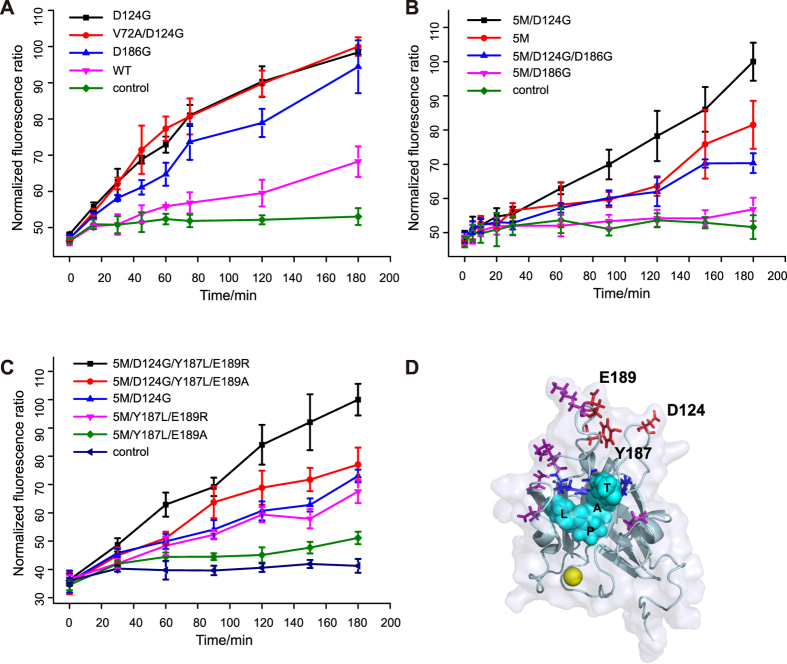
Screening results of the SrtA enzyme. **(A)** Validation of the screening results of the random mutagenesis library. Time lapse measurement of fluorescence ratio was conducted with 1 μM SrtA. 100 μM EGFP and 200 μM cpVenus. Error bars are ± s.d. from the mean of three parallel experiments. **(B)** Combination of beneficial mutations (D124G and D186G) with previously reported 5M mutant. D124G improve the efficiency of the enzyme (black line) while D186G dramatically reduced the efficiency (blue and magenta line). Data represent mean values ±s.d. from three parallel experiments. **(C)** Validation of the screening results of the site-directed saturation mutagenesis library and the rational designed combinations. Mutants harboring mutations at residues Y187 and E189 (magenta and green line) showed improved efficiency. Combination with D124G further improved the efficiency of the mutants (black and red line). Error bars are ±s.d. from three parallel experiments. **(D)** Crystal structure of SrtA in complex with substrate peptide (PDB entry: 2KID). Cyan sphere: Peptide substrate; Blue: Residues involved in substrate-enzyme intermediate formation H120, C184, R197; Magenta: pentamutant; Red: residues evolved in this paper.

We first screened the random mutagenesis library and identified three individual site mutations (V72A, D124G and D186G) as initial hits. Two mutations (D124G and D186G) were proved to be beneficial mutations when compared to WT (Fig. 2A and Supplementary Figure 4A). Interestingly, the D124G mutation in combination with the pentamutant (5M/D124G) resulted in further improvement in activity, as monitored using the FRET based assay, while combination of D186G with the pentamutant (5M/D186G) resulted in reduced activity (Fig. 2B). The D186G mutation was also discovered in the previous yeast display campaign^34^, but not included in their best combination variants, which was consistent with our observations. Not surprisingly, the combination of both D124G and D186G mutations with the pentamutant (5M/D124G/D186G) did not lead to overall improvement in activity (Fig. 2B).

In a complementary approach, we screened the site-saturation mutagenesis library. Residues near the substrates binding groove (Y187, E189, T191, G192, and I199) were mutated on the 5M template. A total of three site-saturation libraries were generated with the combination of Y187 and E189, T191 and G192, or I199 alone (Supplementary Figure 2). As inspired by our finding that D124G mutation improved the efficiency of the enzyme, an additional library was also constructed at residues D124 and R125 on the WT srtA gene (Supplementary Figure 2). Our FRET-based screening against these libraries showed that certain mutations at residues Y187 and E189 enhanced the enzymatic activity, whereas no beneficial mutations were found at residues T191, G192 and I199 (Fig. 2C and Table 1). In particular, two mutants (5M/Y187L/E189R and 5M/Y187L/E189A) exhibited improved enzyme activity than 5M which still displayed beneficial effects when combined with the D124G mutation (Fig. 2C and Table 1, mutants 5M/D124G/Y187L/E89R and 5M/D124G/Y187L/E189A). The combination of mutations at residue Y187 and E189 with the D186G was not beneficial, as demonstrated by another variant bearing Y189R/E189K mutations (5M/Y187R/D189K, Supplementary Figure 4B). Finally, screening of the saturation mutation library at residues D124 and R125 did not yield more efficient mutations than D124G. Taken together, our high throughput screening identified several SrtA variants with improved ligation activity.

### Measurement of the kinetic parameters of the mutants

To better understand how our obtained beneficial mutations enhance SrtA activity, we measured the kinetic parameters of these variants using an established HPLC (High Performance Liquid Chromatography)-based method^36^. The results are summarized in Table 1 and individual plots are shown in Supplementary Figure 5 and 6.

The D124G mutation showed a 1.6-fold increase in k_cat_ and a 1.8-fold decrease in k_mLPETG_ which results in a 3-fold improvement in catalytic efficiency compared to WT. The beneficial effects of D124G mutation were additive to all mutants. The measured parameters for the pentamutant (P94R/D160N/D165A/K190E/K196T) varied slightly from previously reported values^34^, as we observed lower k_cat_ and higher k_mLPETG_ values. Nonetheless, combinations of mutations at residues D124, Y187 and E189 with 5M gave a 1.1 to 4.6 folds improvement in catalytic efficiency. The k_mGGG_ values of the mutants are comparable with that of the 5M, with a slight decrease observed for the 5M/D124G/Y187L/E189R mutant.

As rationalized by previous work, four residues (P94, D160, D165 and K196) of the 5M lie near the substrate binding groove which may improve the enzyme efficiency by affecting substrate binding. This highlights the critical role of this region. The fifth residue of 5M-K190 lies in the middle of the β7/β8 loop. Two of the beneficial mutations that we discovered at Y187 and E189 also lie in the middle region of the β7/β8 loop as K190 does (Fig. 2D). These mutations may affect the substrate binding by changing the flexibility of this loop region. Residue D124 lies in the β4/H2 loop which is also near the substrate binding groove (even though not as close as those four residues in 5M, Fig. 2D). As H120, a residue involved in the substrate-enzyme intermediate formation, also resides in this loop, it is possible that the D124G mutation may change the orientation of the loop to affect the intermediate formation, which leads to improved enzymatic activity.

### Improved protein labeling at both carboxyl and amino terminuses and internal lysine residue side chain

Using our evolved SrtA mutants, we next sought to qualitatively demonstrate labelling of model proteins with small molecular probes. We compared three of our best variants (5M/D124, 5M/Y187L/E189R, 5M/D124G/Y187L/E189R) with the 5M variant which has been shown previously to improve labeling, on both carboxyl and amino terminuses.

**Figure 3:**
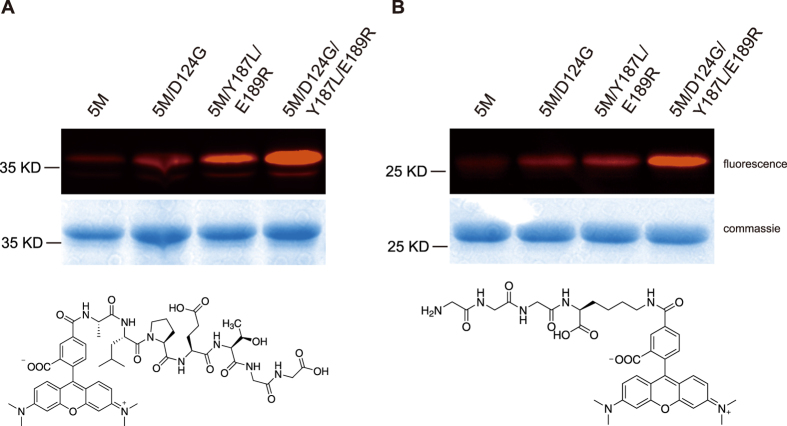
The evolved SrtA improved labeling of model proteins at both the carboxyl and amino terminuses. **(A)** Labeling of model protein GGG-cpVenus with TAMRA-ALPETGG peptide probe. Top: labeling results; bottom: structure of labeling probe TAMRA-ALPETGG. **(B)** Labeling of model protein EGFP-LPETG with GGGK(TAMRA) probe. Top: labeling results; bottom: structure of labeling probe GGGK(TAMRA). This figure only showed the regions of the labeled substrates. Full gel images were shown in Supplementary Figures 14 and 15 with selected regions marked in red boxes.

Model protein cpVenus with N-terminal triglyine residues was first labeled with TAMRA-ALPETGG peptide (Fig. 3A, bottom). Evolved SrtA mutants 5M/D124G, 5M/Y187L/E189R and 5M/D124G/Y187L/E189R showed improved labeling compared to the 5M mutant (Fig. 3A) under the same labeling conditions. Similarly, model protein EGFP with C-terminal LPETG sorting motif was labeled with GGGK(TAMRA) peptide (Fig. 3B, bottom). Elevated efficiency was also observed for these three mutants compared to the 5M mutant (Fig. 3B). Both of the modification sites were confirmed by MS/MS (Supplementary Figure 7).

A recent work reported the non-canonical labeling of proteins with SrtA at lysine residue within the YPK motif^7^. We performed this non-canonical labeling on model protein EGFP bearing either YPKH or YPKN motif in the carboxyl terminus with TAMRA-ALPETGG peptide. Whereas no obvious differences were observed with and without the D124G mutation (Supplementary Figure 8, lane 1 and lane 3 vs. lane 2 and lane 4), mutations at residues Y187 and E189 indeed improved this non-canonical labeling efficiency (Supplementary Figure 8, lane 3 and lane 4 vs. lane 1 and lane2).

These results further demonstrated the enhanced activity of our evolved SrtA variants over the previously reported 5M mutant for labeling the carboxyl and amino terminus of model proteins, as well as for labeling of the internal lysine side chain.

### Improved antibody labeling with evolved SrtA

**Figure 4:**
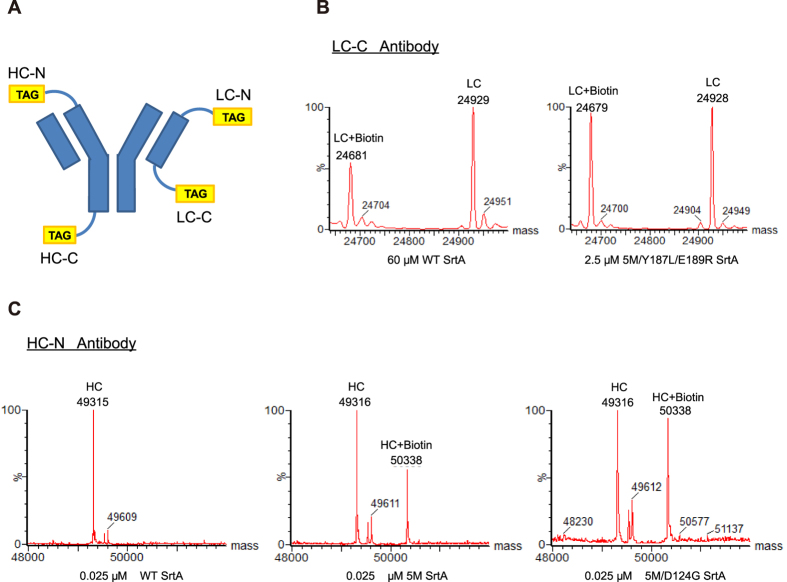
Improved SrtA variants are efficient at labelling an anti-HER2 antibody. **(A)** Four versions of an anti-HER2 antibody were generated with a tag for sortase conjugation on either the N or C-terminus of both heavy and light chains. Corresponding peptide tag sequences are shown in the Methods and Materials section. Each antibody contains identical heavy and light chains, although only one tag is depicted here for simplicity. **(B)** LC/MS trace showing labelling of the light chain C-terminus tagged antibody (LC-C) with either 60 μM of WT SrtA or 2.5 μM of the 5M/Y187L/E189R SrtA variant using GGGY-Lys(Biotin)-NH_2_. **(C)** LC/MS trace comparing labelling of the heavy chain N-terminus tagged antibody (HC-N) with 0.025 μM of either WT, 5M, or 5M/D124G SrtA using Biotin-C6-LELPETGG-NH_2_.

We next performed a more detailed investigation into the utility of our improved sortase variants in performing conjugations on antibodies as we are particularly excited by the possibility of applying these improved sortase variants to generate antibody drug conjugate (ADCs). WT Sortase A and the four improved variants (5M, 5M/D124G, 5M/Y187L/E189R and 5M/D124G/Y187L/E189R) were compared in terms of their efficiency in conjugating biotin as a surrogate toxin payload (see molecular structures in Supplementary Figure 9) onto an anti-HER2 antibody modified to contain a sortase recognition sequence. Four versions of the antibody were made that had a peptide tag placed on either the N or C terminus of either the heavy or light chain of the antibody (Fig. 4A, see Materials and Methods for peptide tag sequences) in order to fully characterize conjugation efficiency across multiple sites. Mass spectrometry was used to determine the degree of conjugation and confirm that a single conjugation took place solely on the tagged chain.

Across the four antibody variants that were tested, the four sortase mutants all had improved efficiency compared to WT sortase (Supplementary Figure 10). Large differences were observed in the amount of sortase required for labelling at each site, and the concentration chosen for comparison at each site was selected to give a range of conjugation efficiencies. In general, we observed that the sortase-mediated reactions required lower concentrations of sortase enzyme for conjugation on the N-terminus compared to the C-terminus of the antibodies, in agreement with the observations of others^37^.

For labelling the N-terminus of the antibody, we found that the 5M/D124G sortase mutant had the highest efficiency on both the light and heavy chain. Interestingly the addition of the D124G mutation seems to synergistically improve the efficiency compared to the 5M variant alone (Fig. 4C). For labelling on the C-terminus of the antibody, we found the 5M/Y187L/E189R variant to be the most efficient. The 5M/Y187L/E189R variant achieved comparable levels of labeling as WT sortase while using 24–40 folds lower concentration of enzyme (0.25 μM vs. 10 μM for the heavy chain, 2.5 μM vs. 60 μM for the light chain) as shown in Fig. 4B. The D124G mutation appeared to be beneficial when combined with the 5M variant for labelling on both the N and C termini, but did not increase efficiency when combined with the 5M/Y187L/E189R variant. The complete results are listed in Supplementary Figure 10 and MS/MS was used to confirm the modification sites (Supplementary Figure 11). Notably, we observed that the kinetic parameters which were determined on peptide substrates did not directly correlate with the labeling efficiency that we observed on the antibodies. We reasoned that this may be due to the differences in the steric hindrance between the peptide substrates and the N- or C- terminus of antibody substrates. It is also possible that some interactions may exist between the specific sequences of the antibody’s N- or C-terminus and the SrtA variants.

### Binding and Internalization of a fluorophore antibody conjugate generated using sortase mediated ligation

In order to demonstrate the utility of these improved sortase variants in making antibody conjugates, we used the 5M/Y187L/E189R variant to generate an anti-HER2 antibody labelled at the C-terminus of the heavy chain with an azide group. Subsequent reaction with a Cy3-dibenzylcyclooctyne (DBCO-Cy3) fluorescent dye using copper free click chemistry resulted in an antibody-fluorophore conjugate (Supplementary Figure 12). We used the Cy3 dye as a surrogate for a typical small molecule cytotoxic payload that would be used for a therapeutic ADC that could be attached using a similar conjugation scheme.

To confirm that the sortase mediated conjugation reaction did not affect the binding of our antibody, we performed a binding ELISA using the N87 Her2 expressing cell line and compared binding of the unmodified HC-C antibody with HC-C antibody after conjugation to Cy3 dye using 5M/Y187L/E189R sortase. As shown in Supplementary Figure 13, similar EC50 values were obtained for the unmodified and conjugated antibodies.

**Figure 5:**
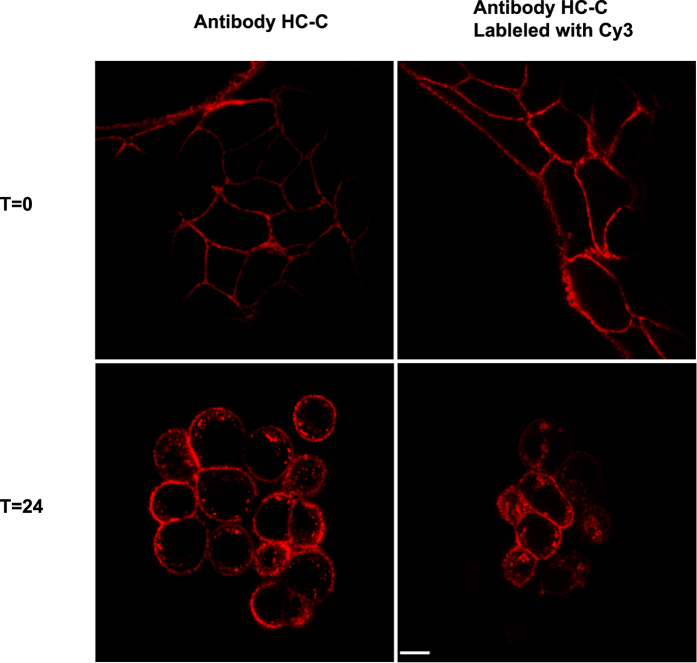
Internalization of anti-HER2 antibody is unaltered by fluorophore conjugation. Confocal imaging of N87 Her2 expressing cells treated with anti-HER2 antibody fluorophore conjugate demonstrate that internalization is not affected by sortase mediated ligation. Her2 expressing N87 cells were treated either with the anti-HER2 HC-C antibody or with HC-C-Cy3 antibody conjugate generated using SrtA mediated ligation. The cells were treated for 45 minutes at 4 °C, washed, and incubated for 24 hours prior to fixation and imaging by confocal microscope. The white scale bar in the bottom right image represents 5 microns.

In addition, we also examined the ability of our anti-HER2 antibody fluorophore conjugate to bind and internalize by confocal microscopy. N87 Her2 expressing cells were incubated with anti-HER2-fluorophore conjugate and showed positive staining on the cell surface as expected. After 24 h of incubation clear internalization was observed (Fig. 5). No differences were observed when compared to the images obtained using the unlabeled HC-C antibody and an anti-human Fc secondary antibody, demonstrating that sortase-mediated labeling did not affect binding or internalization.

### PEGylation using improved variants of Sortase

**Figure 6:**
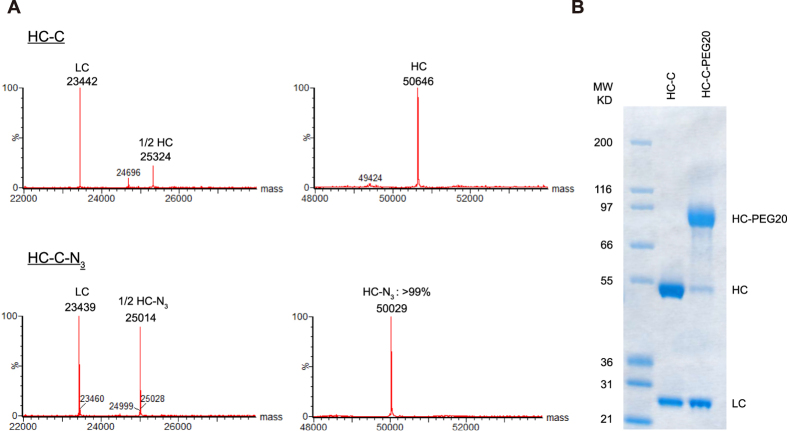
Generating an antibody PEG conjugate using an improved SrtA variant. PEGylation was performed by first conjugating an azide to the HC-C antibody using 1 μM of the 5M/Y187L/E189R sortase variant. (A) LC/MS chromatograms showing the sortase mediated attachment of the azide. **(B)** Subsequent reaction with DBCO-PEG-20 kDa overnight shows the expected shift in molecular weight solely on the antibody’s heavy chain by SDS-PAGE.

We next asked the question as to whether our improved variants could also be employed for polyethylene glycol (PEG) attachment in a highly efficient site-specific manner. Adapting the conjugation scheme used to generate antibody-fluorophore conjugates, we used the improved 5M/Y187L/E189R variant to attach an azide to the C-terminus of the HC-C antibody. As shown in Fig. 6A, conjugation proceeded to completion using just 1 μM of the 5M/Y187L/E189R enzyme. We found that in order to achieve similar levels of conjugation with the azide substrate and WT sortase required enzyme concentrations that were 60 fold higher as those with 5M/Y187L/E189R. Subsequent reaction with a DBCO modified 20 kDa PEG gave the PEGylated construct in high yield, with PEGylation restricted to the heavy chain (Fig. 6B).

## Discussion

Site-specific conjugation strategies are an important technology for generating modified biologics. Enzymatic methods such as the sortase based platform we have described here are inherently dependent on the kinetics of the enzyme. Using a unique FRET based high throughput screening strategy we were able to further improve upon the kinetic properties of SrtA and identify several beneficial mutations that led to improved conjugation efficiency. Subsequent measurement of the kinetic parameters of these mutants validated the importance of these mutations in improving the efficiency of the enzyme.

The improved variants we have identified here can be readily used in a wide-range of conjugation applications. We showed how labeling with our sortase variants improved conjugation efficiency on multiple sites on both proteins and antibodies. The 5M/Y187L/E189R variant we identified was highly effective for conjugations that took place on the C-terminus of either of the antibody chains, while the 5M/D124G variant was superior for conjugations that took place on the N-terminus. Importantly, we demonstrated that all of our variants could achieve conjugation yields similar to the WT enzyme while requiring significantly lower amounts of enzyme. This would be of obvious benefit for commercial scale generation of ADCs or other therapeutic molecules using this conjugation technology.

Interestingly, we observed differences in several variants’ abilities to label the light chain or heavy chain of the antibody, as well as the N or C-terminus. We believe this is likely due to a combination of multiple factors, including differences in their affinity for the LPETG or GGG containing substrates, and differences in the steric accessibility around the tag location of specific sites. The role of steric hindrance is especially apparent when one compares the difference in labelling efficiency seen between the heavy and light chains. For example, in the case of 5M/Y187L/E189R it took a 10 fold higher enzyme concentration to achieve a similar level of conjugation on the C-terminus of the light chain as on the heavy chain, despite having the same C-terminal tag and biotin substrate. As previously mentioned, a prior report also detailed the addition of flexible spacer^35^ element between an antibody and the sortase tag to improve conjugation efficiency, likely due to steric hindrance. When applying sortase mediated conjugation to a novel protein, the availability of multiple variants with differential site preference will allow for screening and applying the optimal variant for a specific site of interest.

Antibody drug conjugates are a specific application where conjugation with sortase could be particularly useful. The efficacy of ADCs is dependent on their ability to internalize efficiently to deliver the cytotoxic drug. We were able to generate fully conjugated antibody-fluorophore conjugates that retained the ability to bind and internalize their target in the HER2-expressing N87 cancer cell line. The high conjugation efficiency observed for small molecules with our improved sortase variants should make it easier to obtain homogenously and completely loaded site-specific ADCs. Importantly we also demonstrated the ability to perform the conjugation at four unique sites on the antibody. Given the important role that site of conjugation can play in the *in vivo* properties of the final conjugate, the ability to effectively conjugate at multiple locations is highly advantageous.

It is worth noting that our improved SrtA variants offer an orthogonal conjugation strategy to other methods for attaching small molecules to antibodies. By combining this method with other approaches, it should be possible to generate ADCs with two different linker payloads which may be expected to better overcome resistance mechanisms if the payloads are from unique classes. In addition, one can envision using one conjugation method to attach a linker payload, and a second method to site specifically attach a fluorescent dye for imaging the trafficking of the ADC. Typically for these applications, attachment of the dye is done through non-specific labelling, which has the potential to interfere with binding and/or trafficking for example if the dye is attached in the antibody complementarity determining regions (CDRs). Having a site specific method for attaching the dye which is orthogonal to the method for attaching the linker payload would help to avoid these potential issues.

PEGylation is another application for our improved sortase variants and is a well-established method for extending the half-life of biologics. Controlling the site of attachment of the PEG to the protein of interest is often critical, as the PEG can potentially block sites required for activity, resulting in reduced efficacy. Site-specific methods such as sortase mediated ligation offer obvious benefits in such situations. In our case we found that our improved variant 5M/Y187L/E189R could be used to attach PEG to the engineered site with high efficiency in a site-specific manner. The conjugation required a 60 fold lower amount of enzyme when compared with what was required for the WT enzyme.

Unlike the N-terminus for which several site-specific approaches to PEGylation such as reductive amination or serine/threonine oxidation are known, the chemical reactivity of the C-terminus does not generally allow for it to be targeted in a site-specific manner. As a result, PEGylation of the C-terminus is typically performed by introduction of a cysteine residue, which has the potential for undesired reactions if the native protein contains an unpaired cysteine. This can be especially problematic as attachment of PEG at the incorrect location can cause loss of activity. In this regard, the site specific nature of enzymatic conjugation using SrtA offers a clear benefit.

The improved SrtA variants described here should find general use in conjugation strategies for making both protein and antibody conjugates.

## Materials and Methods

### Reagents

The TAMRA-ALPETGG, GGGK(TAMRA) and Abz-LPETGK(Dnp)-CONH_2_ were synthesized by GL Biochem (Shanghai) Ltd. GGG was purchased from Sigma-Aldrich. The Biotin-C6-LELPETGG-NH_2_, GGGY-Lys(Biotin)-NH_2_, and GGG-Lys(N3)-NH_2_ reagents were synthesized by CPC Scientific. The DBCO-Cy3 fluorescent dye was obtained from Click Chemistry Tools (A140-5).

### Validation of FRET based platform

100 μM EGFP and 200 μM cpVenus was reacted in the presence or absence of 1 μM WT SrtA enzyme at 37 °C for 2 hrs. Reaction mixture was then excited at 435 nm and emission from 450 nm to 600 nm was collected with Fluorescence Spectrophotometer (Agilent Technologies). For validation as high throughput readout, 100 μM EGFP and 200 μM cpVenus was reacted in the absence or presence of 50 nM WT or 5M SrtA enzyme at 37 °C for 2 hrs. Reaction mixture was excited at 435 nm and emission at 475 nm and 525 nm was collected with Synergy H4 Hybrid Microplate Reader (BioTek). Fluorescence ratio at 525 nm and 475 nm was calculated as readout. For each individual experiment, the highest fluorescence ratio was normalized to 100.

### Generation of Sortase A random mutagenesis library

Random mutagenesis library of the WT sortase A gene was generated by error-prone PCR (F: CGCGGATCCAAACCACATATC, R: CCGGAATTCTTATTTGACTTC). A plasmid containing WT SrtA gene was used as template. The PCR conditions were adjusted by reducing dATP concentration to 20 μM and adding 200 μM dITP. The restriction endonuclease (BamHI/EcoRI) digested sortase A gene fragment was then ligated to pet28a vector and transformed into DH10B competent cell. All transformants were harvested. Plasmids were collected and transformed into BL21(DE3) competent cell for library expression. Site-directed saturation mutagenesis library of the pentamutant sortase A gene was generated with normal site mutation PCR method. Primers were as below. PCR reaction mixture was directly transformed into DMT competent cells. All transformants were harvested, plasmids were collected and transformed into BL21(DE3) competent cell for library expression.

Y187/E189-F: ATTACTTGTGATGATNNKAATNNKGAGACAGGCG

Y187/E189-R: MNNATTMNNATCATCACAAGTAATTAATGTTAAT

T190/G191-F: GATGATTACAATGAANNKNNKGGCGTTTGGG

T190/G191-R: MNNMNNTTCATTGTAATCATCACAAGTAATT

I199-F: TGGGAAACACGTAAANNKTTTGTAGCTA

I199-R: MNNTTTACGTGTTTCCCAAACGCCTGTC

D124/R125-F: GGACACACTTTCATTNNKNNKCCGAACTATC

D124/R125-R: MNNMNNAATGAAAGTGTGTCCTGCAATTGAA

### High throughput screening

BL21(DE3) transformants of the SrtA mutagenesis library were first cultured overnight at 37 °C in 96-well plates with 1 mL LB medium containing 100 μg/mL kanamycin. Overnight bacteria was then re-inoculated into new 96-well plates with a ratio of 1:100 dilution in 1 mL volume and were grown at 37 °C for about 3 hr till OD_600_ reached about 0.6. 1 mM IPTG was then added to the medium to induce the expression of SrtA mutants at 37 °C for 3 hr. Induced cells were harvested by centrifugation at 4,700 rpm for 10 min and lysed with 200 μL lysis buffer (30 mM Tris, 15 mM NaCl, 5 mM CaCl_2_, 0.15 mg/mL lysozyme, 1% triton x-100, pH 7.4) at 37 °C for 2 hr. Cell lysates were centrifuged at 4,700 rpm for 10 min and pellets were discarded. Sortase-mediated ligation was conducted in 96-well plate between 100 μM EGFP-LPETG and 200 μM GGG-cpVenus in a 200 μL total volume in reaction buffer (30 mM Tris, 15 mM NaCl, 5 mM CaCl_2_, pH 7.4) in the presence of 50 μL supernatant of cell lysate. Reaction was preceded at 37 °C for 2 hrs and emission of the reaction mixture at wavelength 525 nm and 475 nm was then measured with hybrid reader with excitation at 435 nm. Fluorescence ratio was calculated and that mutants with higher fluorescence ratio than WT SrtA entered the next round of screening. All conditions were strictly controlled to ensure the expression levels were uniform among SrtA variants. The expression levels of randomly selected variants were also examined by SDS-PAGE and western blotting analysis as a further quality control procedure (Supplementary Figure 3B). The expression variations can be controlled within 5% in our experimental settings.

### Validation of evolved SrtA mutants

For validation of evolved SrtA mutants with purified proteins, time lapse fluorescence ratio was measured. SrtA mediated ligations in Fig. 2A and Supplementary Figure 4A were conducted with 1 μM SrtA, 100 μM EGFP and 200 μM cpVenus. SrtA mediated ligations in Fig. 2B,C and Supplementary Figure 4B were conducted with 50 nM SrtA, 100 μM EGFP and 200 μM cpVenus. For each individual experiment, the highest fluorescence ratio was normalized to 100.

### Measurement of kinetic parameters

Kinetic parameters for WT and variant SrtA were determined with the previously reported methods^35^. SrtA catalyzed reaction between Abz-LPETGK(Dnp)-CONH_2_ and GGG-COOH was measured at 37 °C with reaction time varied from 90 seconds to 15 minutes in buffer 30 mM Tris-HCl, 15 mM NaCl, 5 mM CaCl_2_, pH 7.4. The SrtA concentration varied from 25 nM to 1000 nM. To determine K_cat_ and K_mLPETG_, the concentration of GGG-COOH peptide was settled at 10 mM with Abz-LPETGK(Dnp)-CONH_2_ concentration range from 100 μM to 8 mM. To determine K_mGGG_, the concentration of Abz-LPETGK(Dnp)-CONH_2_ was settled at 1 mM with GGG-COOH concentration range from 100 μM to 20 mM. The reaction was terminated by addition of 1/2 volume of 1 M HCl into the reaction mixture. The mixture was then subjected to UPLC system (ACQUITY UPLC Peptide BEH C18 Column, 130 Å, 1.7 μm, 2.1 mm ∗ 150 mm, Waters). The extent of the reaction was determined by measuring the absorbance of Dnp moiety at 355 nm. Reaction velocity was represented by enzyme turnover per second and fitted to Michaelis-Menten equation with OriginPro 8.5 software to determine parameters. Error bars were determined from at least three individual experiments.

### Sortase mediated labeling of model fluorescent proteins with peptide-fluorophore conjugates

Model fluorescent proteins labeling in Fig. 3 and Supplementary Figure 8 was conducted with 20 μM model protein, 200 μM corresponding labeling probe and 50 nM SrtA in Tris-HCl buffer (20 mM Tris, 500 mM NaCl, 5 mM CaCl_2_, pH 7.4) at 37 °C for 15 minutes. Fluorescent and commassie brilliant blue stained gels were acquired with ChemiDoc and processed with Image Lab software.

### Sortase Mediated Biotinylation of Antibodies

The sortase-mediated biotinylation reaction of the antibodies with a tag on the N-terminus was performed using 2 mM Biotin-C6-LELPETGG-NH_2_, 20 μM N-terminal antibody, 300 mM Tris, 150 mM NaCl, 5 mM CaCl_2_, pH 7.5, and sortase mutants with the concentration varying from 60 μM to 0.01 μM at room temperature for 3 hours, in a 20 μl reaction volume. The reaction was diluted 10-fold in 300 mM Tris, 150 mM NaCl, 5 mM CaCl_2_, pH 7.5 and reconcentrated using a Vivaspin® 500 centrifugal concentrator with a 10,000 kDa MWCO to a final volume of 20 μl to stop the reaction. The same reaction conditions were used with the antibodies containing a C-terminal tag, with the exceptions of using 2 mM GGGY-LysBiotin-NH_2_ and a concentration range of sortase mutants from 60 μM to 0.25 μM. Samples were analyzed using LC/MS (described below). The conjugation on the N-terminus was readily detected by an increase in mass of 1022 Da. The C-terminal reaction was similarly detected by a change of −249 Da.

### Generation of an Antibody-Fluorophore conjugate using Sortase

The heavy chain C-terminal tagged antibody was conjugated to GGG-Lys(N3)-NH_2_ via sortase-mediated ligation in a total volume of 500 μl using 20 μM antibody, 1 μM of the 5M/Y187L/E189R sortase mutant, 2 mM GGG-Lys(N3)-NH_2_, and sortase buffer (300 mM Tris, 150 mM NaCl, 5 mM CaCl_2_, pH 7.5) at room temperature overnight. The reaction was stopped by diluting 10-fold in sortase buffer and unreacted azide removed using a Vivaspin® 500 Centrifugal Concentrator with a 10,000 kDa MWCO. To generate the antibody-fluorophore conjugate, the antibody-azide was concentrated to 33 μM and 10 molar equivalents of DBCO-Cy3 were added and allowed to react overnight at room temperature. The reaction was stopped using a vivaspin 500 centrifugal concentrator as previously described to remove unreacted DBCO-Cy3. The Cy3 conjugate was purified by size-exclusion chromatography on a SuperDex200 increase 10/300 GL column in PBS, pH 7.2. The main peak was collected and analyzed using LC-MS.

### Generation of an Antibody-PEG conjugate using Sortase

The HC-C antibody was modified to contain an azide using the 5M/Y187L/E189R sortase mutant and the same protocol outlined above for generation of the antibody-fluorophore conjugate. The antibody-azide conjugate was then reacted with 10 molar equivalents of DBCO-mPEG, 20 kDa (Click Chemistry Tools, A120) overnight at room temperature. The sample was spun in a vivaspin 500 centrifugal concentrator to remove excess PEG and then analysed on a 4–12% BisTris NuPAGE gel with MOPS running buffer. 3 ug of each sample was reduced, boiled and loaded per lane.

### Mass Spectrometry Analysis

Samples were prepared by deglycosylating the antibody using PNGase F (New England Bio Labs) for 2 hours at 37 °C followed by reduction with TCEP. LC/MS analysis was performed on an Aglient 1100 capillary HPLC coupled with Waters Xevo G2 Q-TOF mass spectrometer. 3 μg of sample was injected on a Waters BEH300 C4 column (1.0 mm × 50 mm, 1.7 μm, maintained at 80 °C). 0.5% trifluoroacetic acid (Fisher Scientific) was used as Buffer A and acetonitrile with 0.5% triflouroacetic acid (Fisher Scientific) was used as Buffer B. Samples were eluted using a gradient of 2% Buffer B for 6 minutes, followed by 12% B to 50% B over 24 minutes with a flow rate of 65 μL/min. Mass spectrometric detection was carried out in positive, sensitivity mode with capillary voltage set at 3.3 kV. Data analysis was performed with MaxEnt 1 function in MassLynx and intensities were used to determine conjugation efficiency.

### Cell-based ELISA

HER2-expressing N87 cells were seeded in 96 well plates at 50,000 cells per well and cultured overnight. Cells were blocked and incubated with primary antibody (unmodified and Cy3 modified HC-C antibody, respectively) for 1 hr. Cells were washed with PBS and incubated with anti-human HRP secondary. Sureblue reagent was used for detection and PRISM analysis software was used to calculate binding curves.

### Confocal Imaging

Confocal microscopy was used to determine antibody internalization in HER2-expressing N87 cells. Cells were grown in incubator overnight at 37 °C, 5% CO_2_. Cells were washed with PBS and incubated with antibody (10 μg/ml) for 45 min at 4 °C to initiate binding. Unbound antibody was removed by washing with PBS then cells were returned to incubator in pre-warmed, complete RPMI medium. Cells were incubated for 24 hours then washed and fixed. To image internalization of unlabeled antibody, control samples were permeabilized and stained for 45 minutes with anti-human Fc fluorophore secondary antibody. Cells were washed and imaged on a Nikon Eclipse TE 2000-U confocal microscope.

### Antibody Sequences

The anti-HER2 antibodies used in this study are based on the described huMab4D5 antibody (*Proc. Natl. Acad. Sci. USA* Vol. 89, pp. 4285–4289, May 1992). The tagged versions of the antibody were made by directly appending the following tag sequences to either the light or heavy chain at either the N or C terminus as underlined below. Light Chain C-Terminal-Tag: LIGHT CHAIN-GGLPETGGHHHHHH; Light Chain N-Terminal-Tag: GGG-LIGHT CHAIN; Heavy Chain C-Terminal-Tag: HEAVY CHAIN-GGLPETGGHHHHHH; Heavy Chain N-Terminal-Tag: GGG-HEAVY CHAIN.

### Generation of Tagged Antibodies

The anti-HER2 antibodies were expressed in HEK-293 Freestyle cells (Invitrogen) and transfected with IgG expression plasmids in shake flask culture with Freestyle media (Invitrogen) following the manufacturer’s recommendations for transfection and growth conditions. Antibodies were purified in two steps first by affinity chromatography using a Mabselect sure column followed by size-exclusion chromatography on a Superdex 200 column. Following purification antibodies were found to be greater than 99% pure by analytical SEC.

## Additional Information

**How to cite this article**: Chen, L. *et al.* Improved variants of SrtA for site-specific conjugation on antibodies and proteins with high efficiency. *Sci. Rep.* **6**, 31899; doi: 10.1038/srep31899 (2016).

## Supplementary Material

Supplementary Information

## Figures and Tables

**Table 1:** Kinetic parameters of the SrtA variants.

Mutants	Mutations	K_cat_, s^−1^	K_mLPETG_, mM	K_cat_/K_mLPETG_, M^−1^ s^−1^	K_mGGG_, μM
WT		1.1 ± 0.1	7.1 ± 0.9	159 ± 25	71 ± 8
5M	P94R/D160N/D165A/K190E/K196T	3.7 ± 0.2	1.0 ± 0.1	3622 ± 537	2779 ± 610
5M/D124G	P94R/D124G/D160N/D165A/K190E/K196T	16.5 ± 0.7	1.8 ± 0.2	9305 ± 931	2833 ± 742
D124G	D124G	1.8 ± 0.2	3.9 ± 0.7	472 ± 99	247 ± 40
5M/Y187R/E189K	P94R/D160N/D165A/Y187R/E189K/K190E/K196T	5.2 ± 0.6	2.8 ± 0.5	1868 ± 400	1412 ± 138
5M/D124G//Y187R/E189K	P94R/D124G/D160N/D165A/Y187R/E189K/K190E/K196T	7.9 ± 0.6	1.4 ± 0.2	5519 ± 901	1981 ± 316
5M/Y187L/E189A	P94R/D160N/D165A/Y187L/E189A/K190E/K196T	2.9 ± 0.2	0.7 ± 0.1	4094 ± 748	5580 ± 1204
5M/D124G//Y187L/E189A	P94R/D124G/D160N/D165A/Y187L/E189A/K190E/K196T	15.9 ± 1.7	1.5 ± 0.3	10405 ± 2451	2004 ± 265
5M/Y187L/E189R	P94R/D160N/D165A/Y187L/E189R/K190E/K196T	6.3 ± 0.3	0.55 ± 0.06	11425 ± 1315	2367 ± 610
5M/D124G//Y187L/E189R	P94R/D124G/D160N/D165A/Y187L/E189R/K190E/K196T	22.2 ± 1.3	1.3 ± 0.2	16722 ± 2329	1700 ± 420
