# Supplementary material for: Improved variants of SrtA for site-specific conjugation on antibodies and proteins with high efficiency
Source: Sci Rep. 2016 Aug 18;6:31899. doi: 10.1038/srep31899 (PMC4989145; doi:10.1038/srep31899)
Supplement: Supplementary Information [file srep31899-s1.doc]

**SUPPLEMENTARY INFORMATION**

**Improved variants of SrtA for site specific conjugation on antibodies and proteins with high efficiency**

Long Chen, Justin Cohen, Xiaoda Song, Aishan Zhao, Zi Ye, Christine J. Feulner, Patrick Doonan, Will Somers, Laura Lin^★^ & Peng R. Chen^★^

^★^ Corresponding authors: Laura Lin, Peng R Chen

E-mail: Laura.Lin@pfizer.com, pengchen@pku.edu.cn


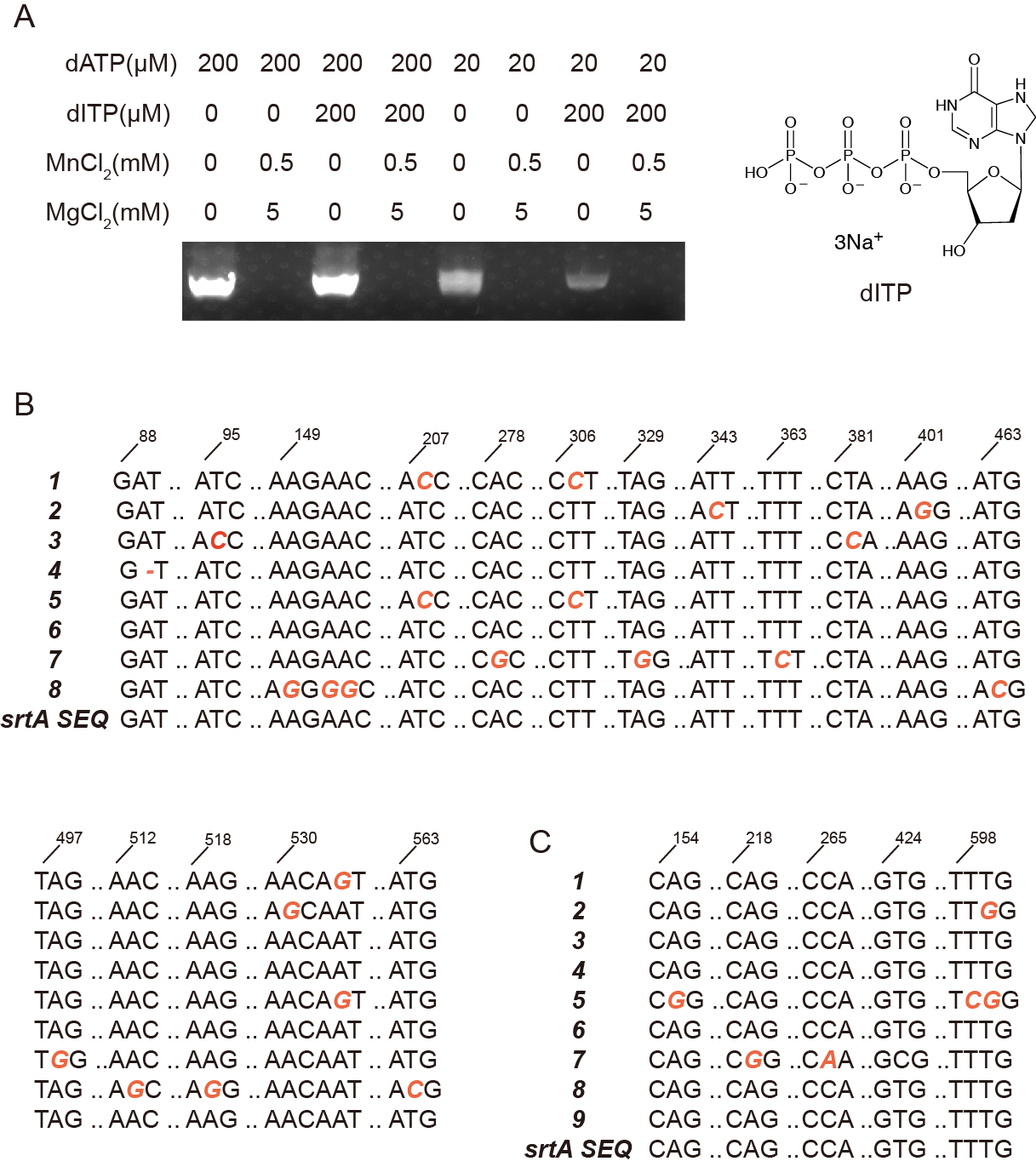


**Figure S1. Conditions of the error-prone PCR and the sequencing results.** A. Validation of the error-prone PCR conditions (left) and the structure of dITP (right); B. Sequencing results with the conditions described in Figure S1A lane 7. This gene library was utilized for the directed evolution; C. Sequencing results with the condition described in Figure S1A lane 3.


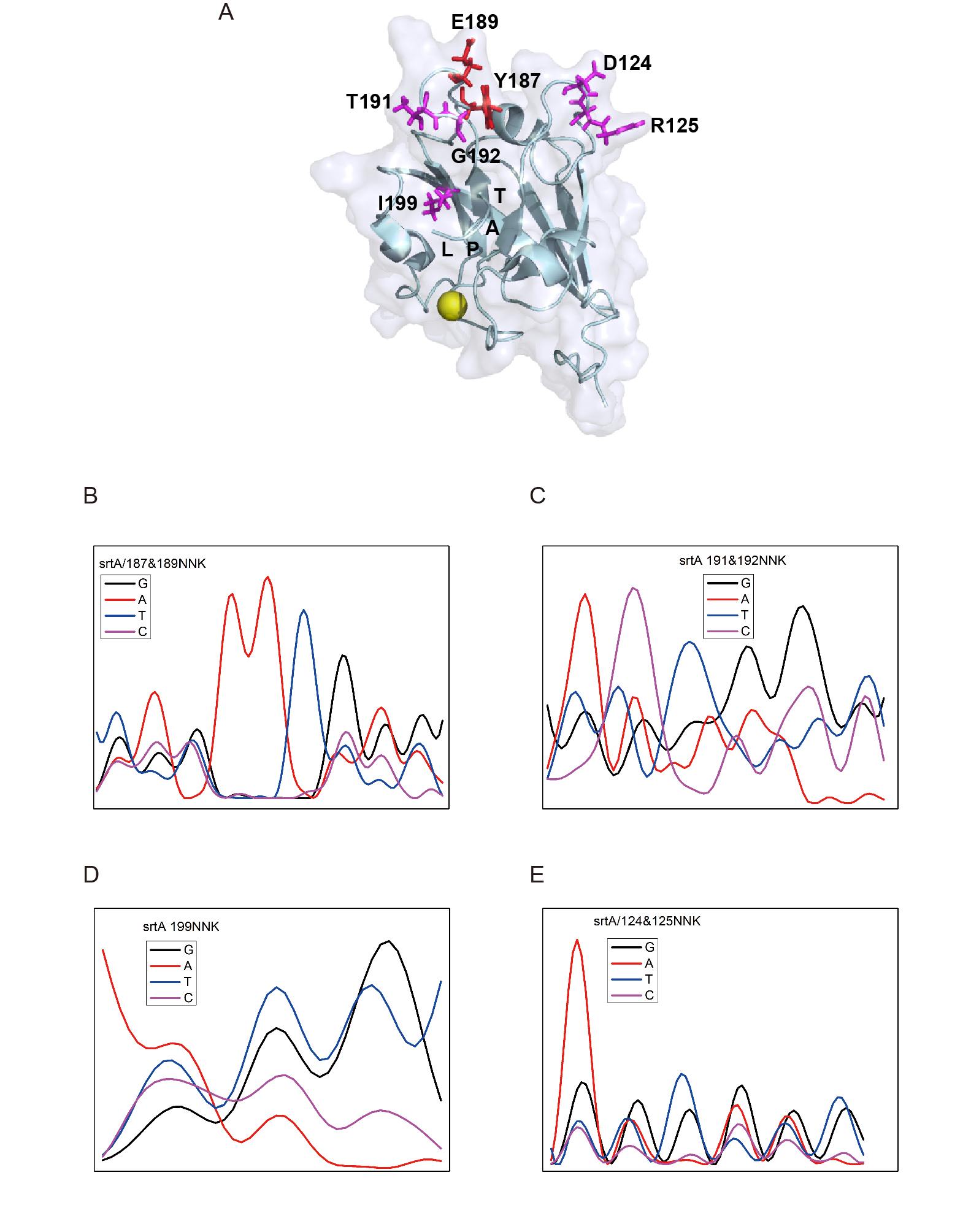


**Figure S2. Selected sites to construct site-saturation mutagenesis library.** A. Overview of the selected sites (PDB entry: 2KID); B-E: Sequencing results of the site-saturation mutagenesis libraries. Site-saturation mutagenesis on residues Y187, E189, T191, G192 and T199 was performed on the pentamutant gene. Site-saturation mutagenesis on residues D124 and R125 was performed on the WT SrtA gene.


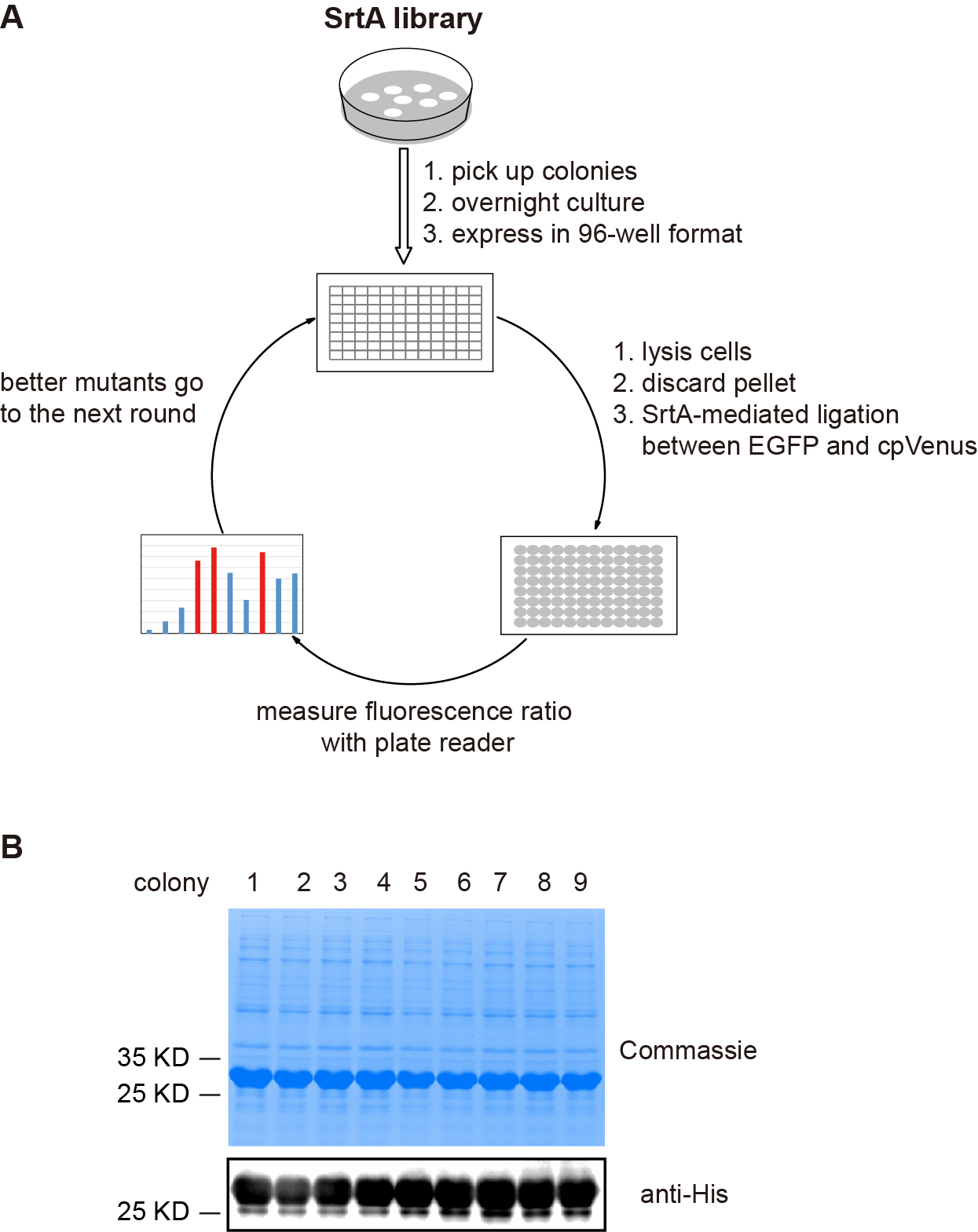


**Figure S3.** A) Designed procedures for high through-put screening; B) Monitoring library expression levels with randomly selected colonies by SDS-PAGE and western blotting.


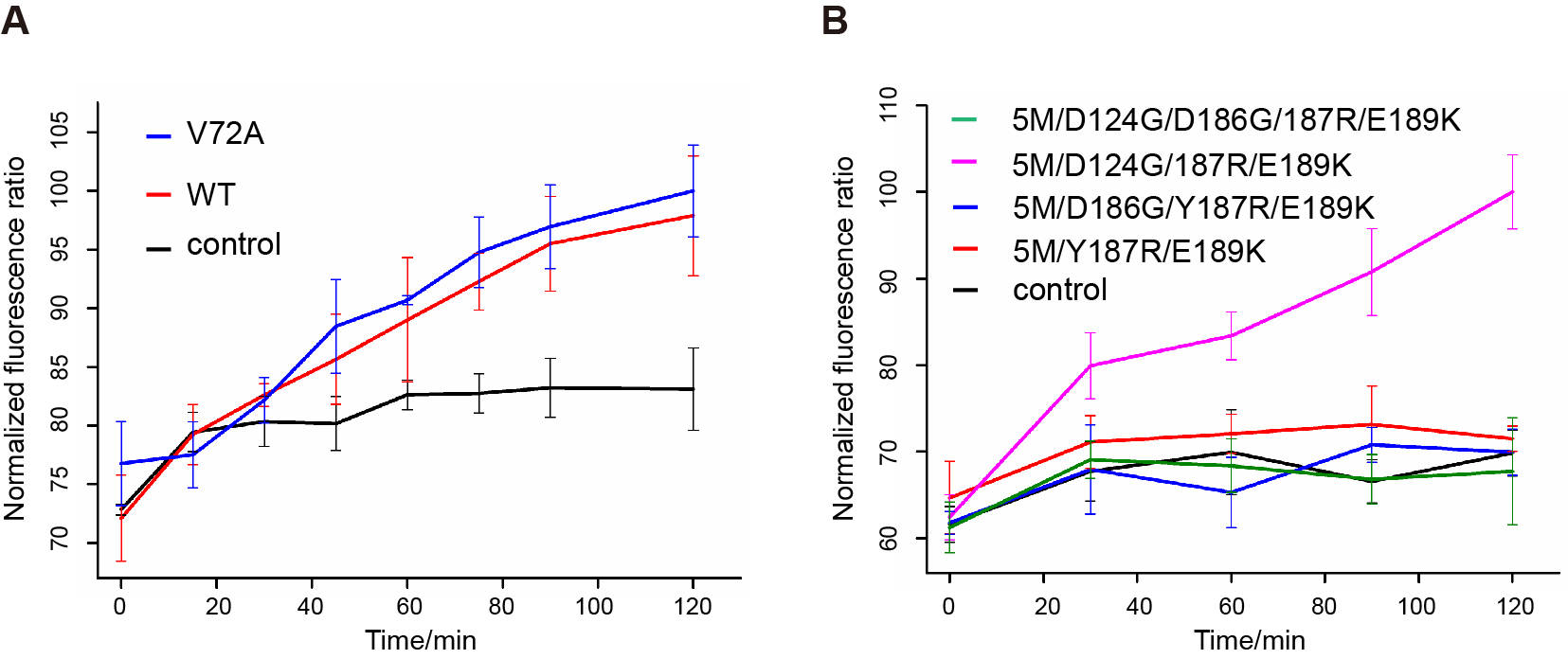


**Figure S4.** A) Validation of the V72A mutation; B) Validation of the combination effect of D124G and D186G mutations with mutations at Y187 and E189.


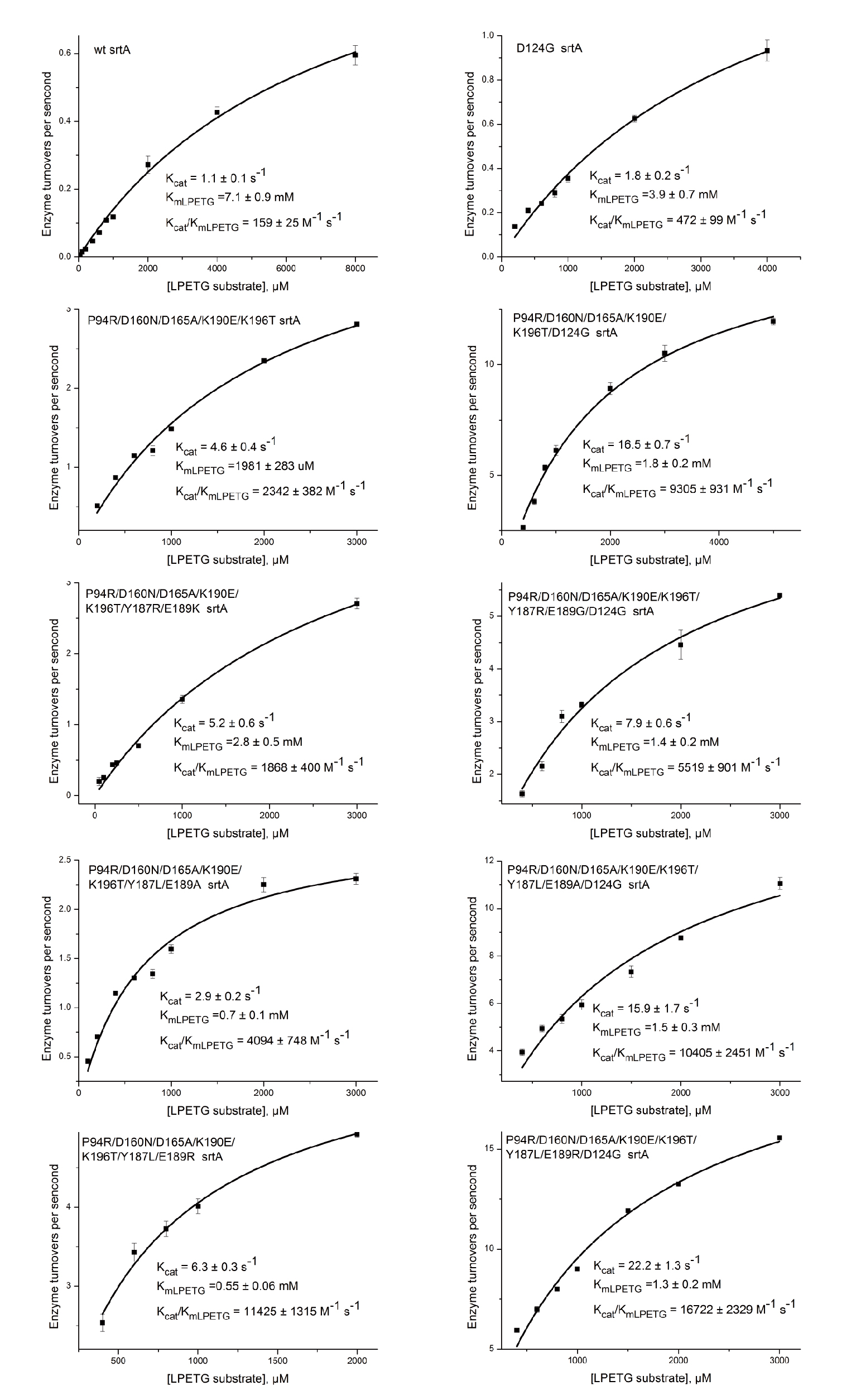


**Figure S5. Plots to determine K_cat_ and K_mLPETG_.**


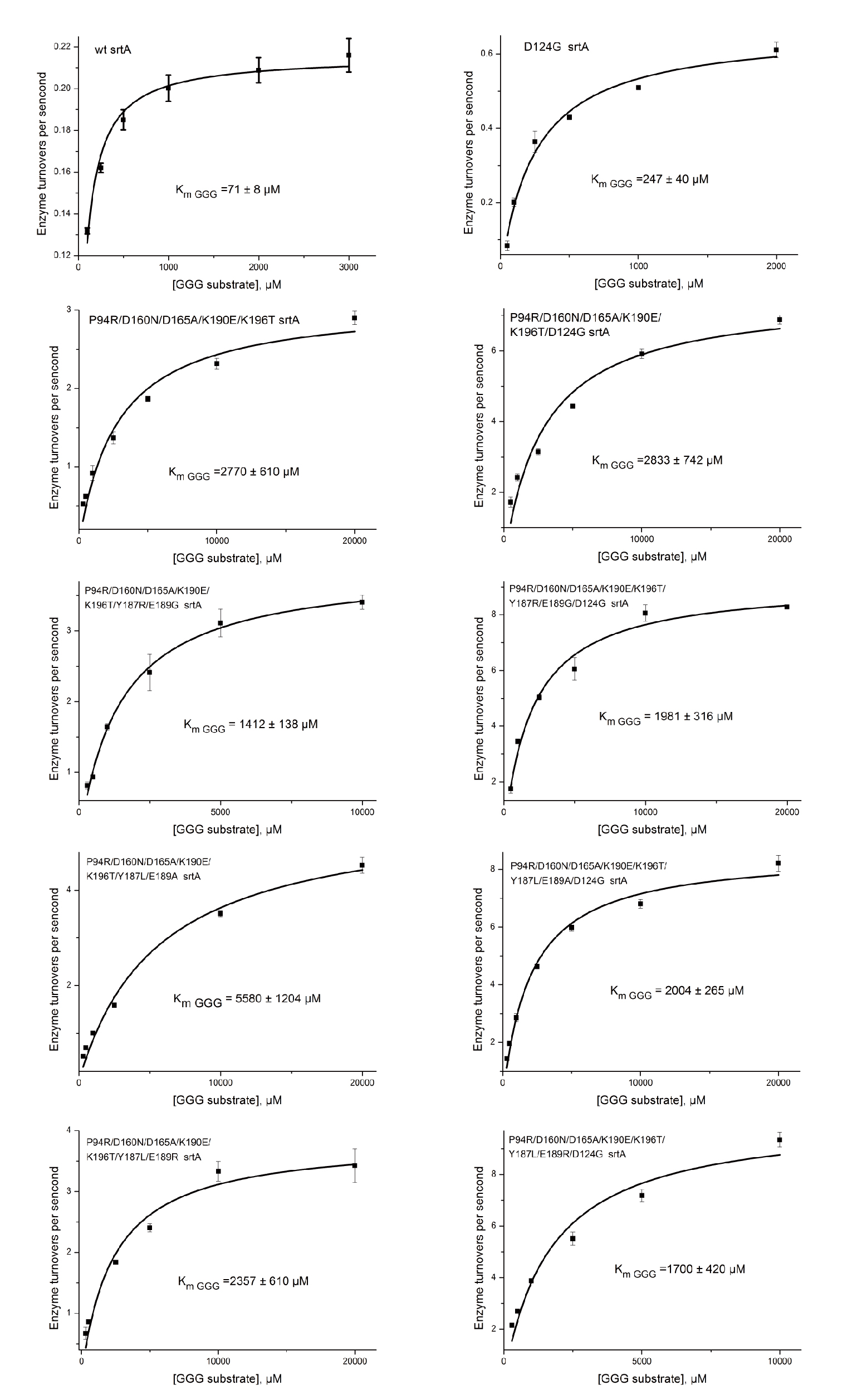


**Figure S6. Plots to determine K_mGGG_.**

**
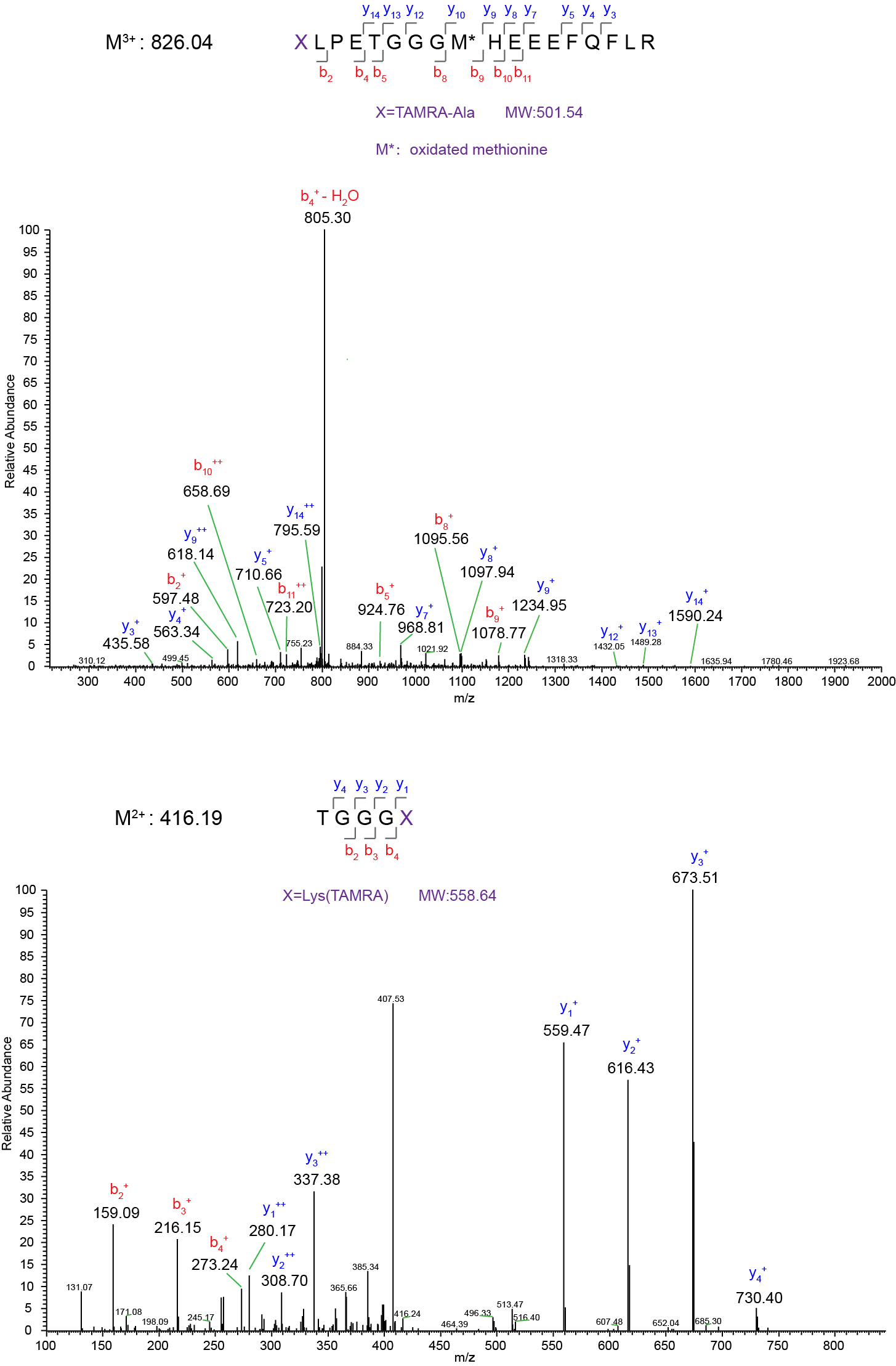
**

**Figure S7. MS/MS spectra of identified target peptides from Figure 3A lane 4(top, digested with trypsin protease) and Figure 3B lane 4 (bottom, digested with Glu-C protease).**


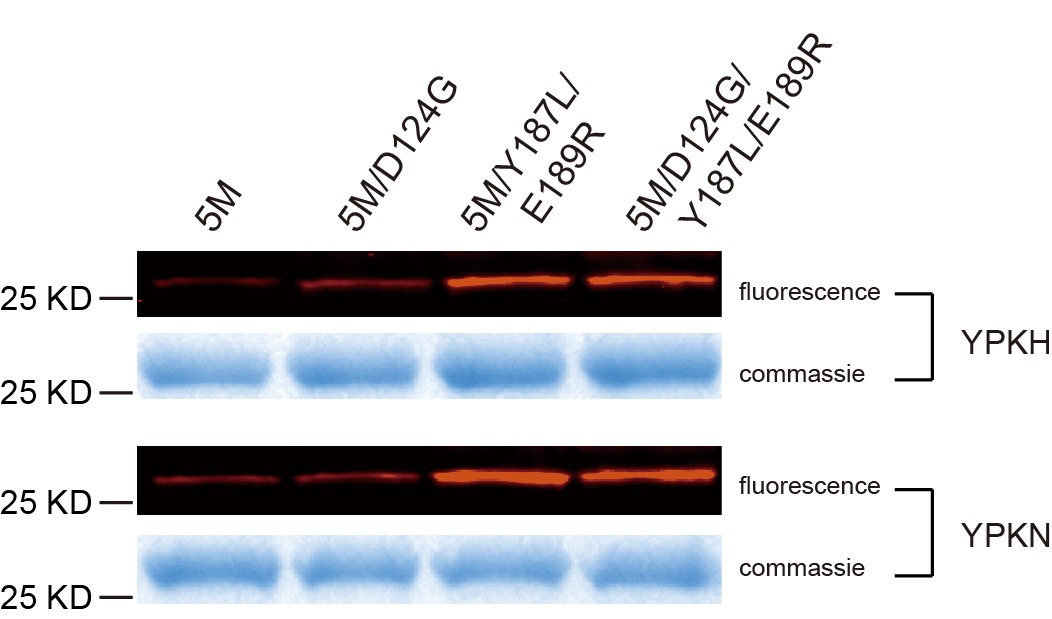


**Figure S8. Improved labeling of internal lysine residue side chain within specific sequence YPKH (top) and YPKN (bottom).** Images represented the regions of the substrates EGFP. Corresponding full images were presented in Supplementary Figure 16. Selected regions were marked in red boxes.


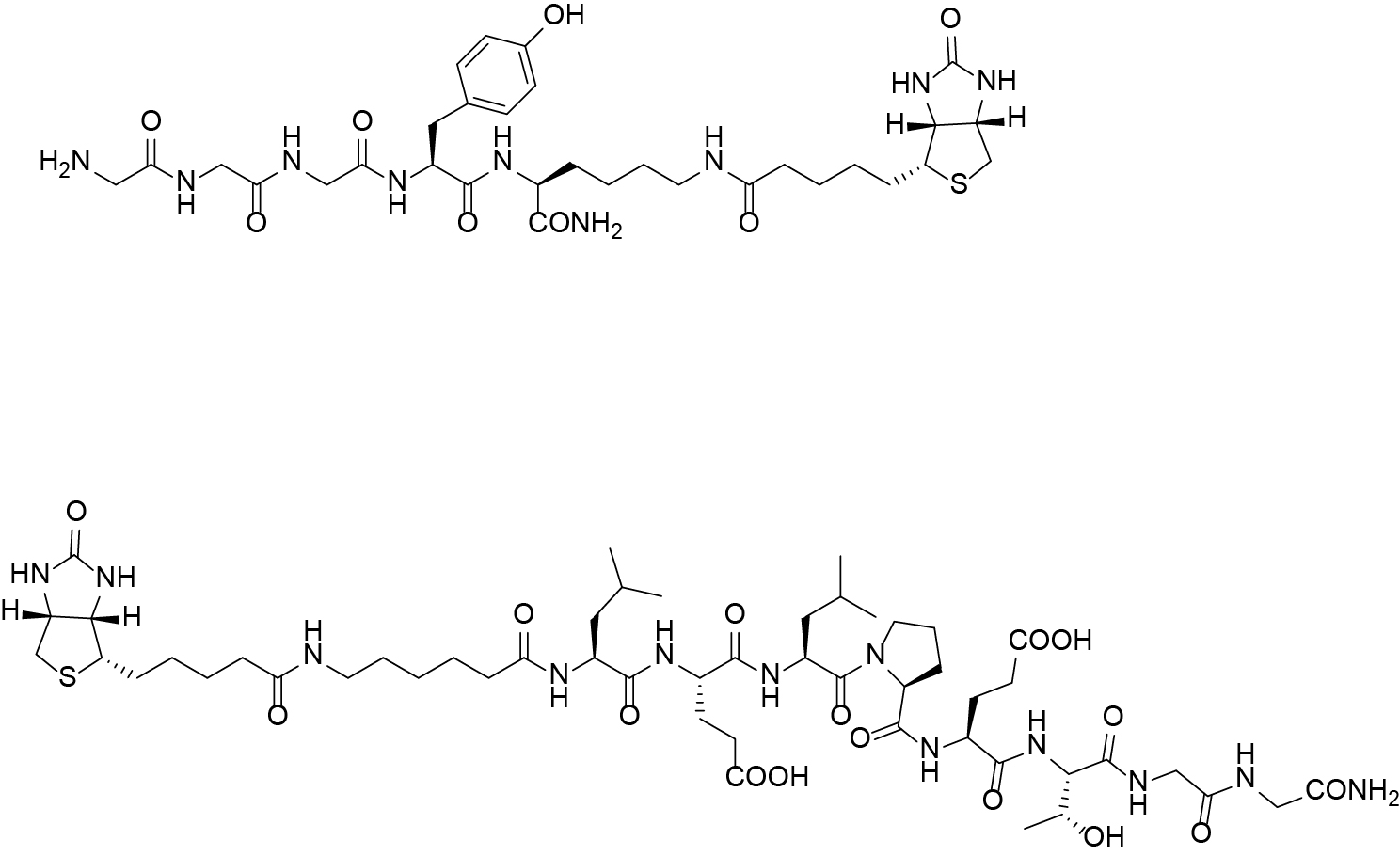


**Figure S9. Molecular structures of the biotin molecules used as surrogate toxin to label antibodies.** Top: GGGY-Lys(Biotin)-NH_2_; bottom: Biotin-C6-LELPETGG-NH_2_.

| **Antibody** | **Sortase** | **Sortase concentration (uM)** | **Product % by LC/MS** |
| --- | --- | --- | --- |
| HC-N | WT | 0.01 | 1.44% |
|  | 5M | 0.01 | 34.83% |
|  | 5M/D124G | 0.01 | 49.71% |
|  | 5M/Y187L/E189R | 0.01 | 3.49% |
|  | 5M/D124G/Y187L/E189R | 0.01 | 5.77% |
| LC-N | WT | 0.025 | 1.09% |
|  | 5M | 0.025 | 19.35% |
|  | 5M/D124G | 0.025 | 87.23% |
|  | 5M/Y187L/E189R | 0.025 | 6.31% |
|  | 5M/D124G/Y187L/E189R | 0.025 | 5.30% |
| HC-C | WT | 10 | 64.42% |
|  | 5M | 0.25 | 9.38% |
|  | 5M/D124G | 0.25 | 19.40% |
|  | 5M/Y187L/E189R | 0.25 | 42.43% |
|  | 5M/D124G/Y187L/E189R | 0.25 | 33.62% |
| LC-C | WT | 60 | 26.75% |
|  | 5M | 2.5 | 15.95% |
|  | 5M/D124G | 2.5 | 33.64% |
|  | 5M/Y187L/E189R | 2.5 | 41.56% |
|  | 5M/D124G/Y187L/E189R | 2.5 | 22.25% |

**Figure S10. Comparison of antibody labelling using SrtA variants.** WT SrtA was compared to 5M, 5M/D124G, 5M/Y187L/E189R, and 5M/D124G/Y187L/E189R for labelling four variants of an anti-HER2 antibody with the peptide tag placed on either the N or C terminus of the heavy or light chain. The concentration of SrtA used was chosen to give a range of efficiency in order to compare variants. Product % was calculated as the ratio of the signal intensity of the product mass versus the signal intensity of the unmodified mass as determined by LC/MS in a single experiment.


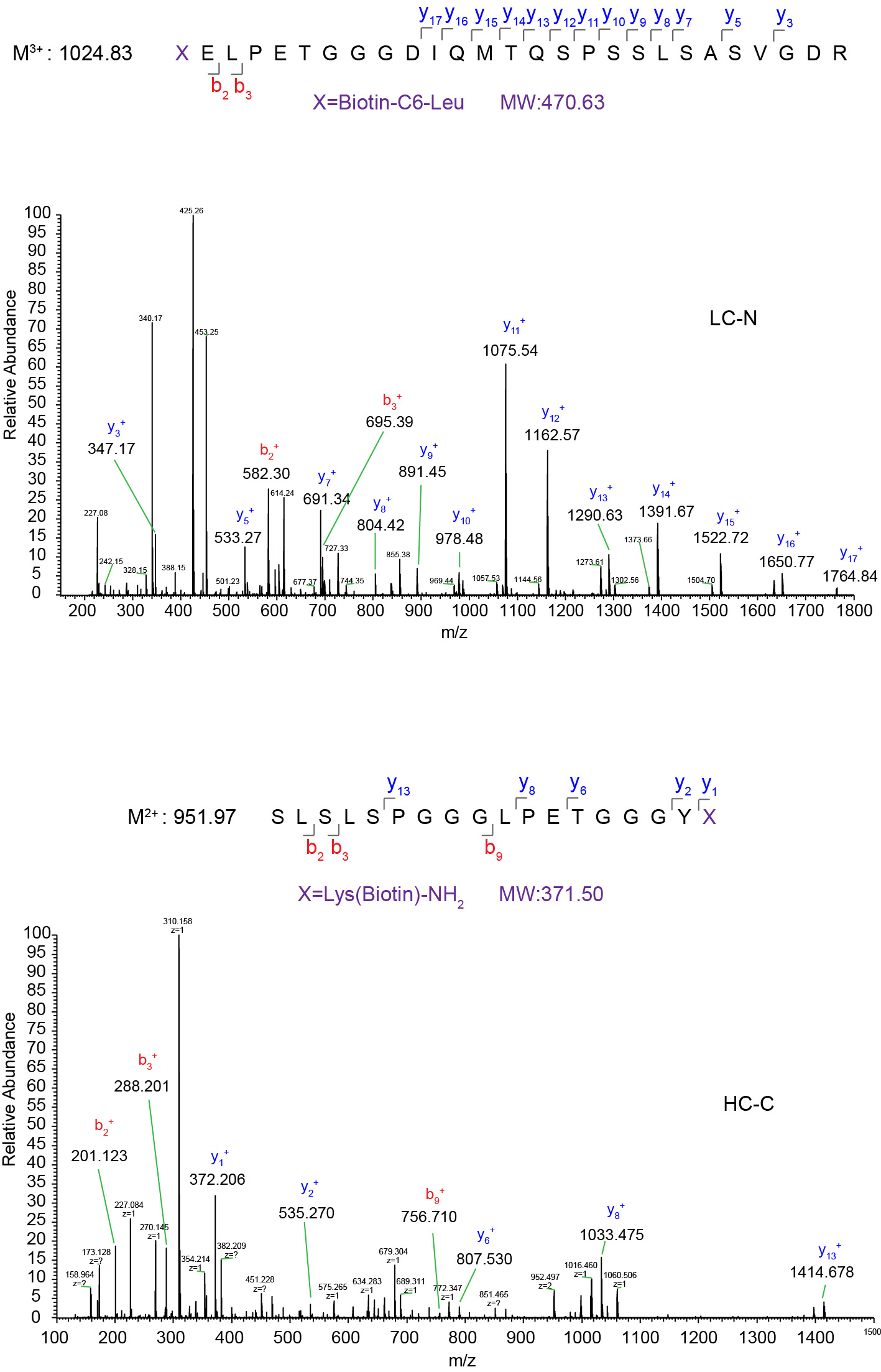


**Figure S11. MS/MS spectra of identified target peptides from antibodies labelling.** Top: identified peptide from light chain N-terminus labelled with 5M/D124G SrtA; Bottom: identified peptide from heavy chain C-terminus labelled with 5M/Y187L/E189R SrtA. Antibodies were digested with trypsin protease.

**
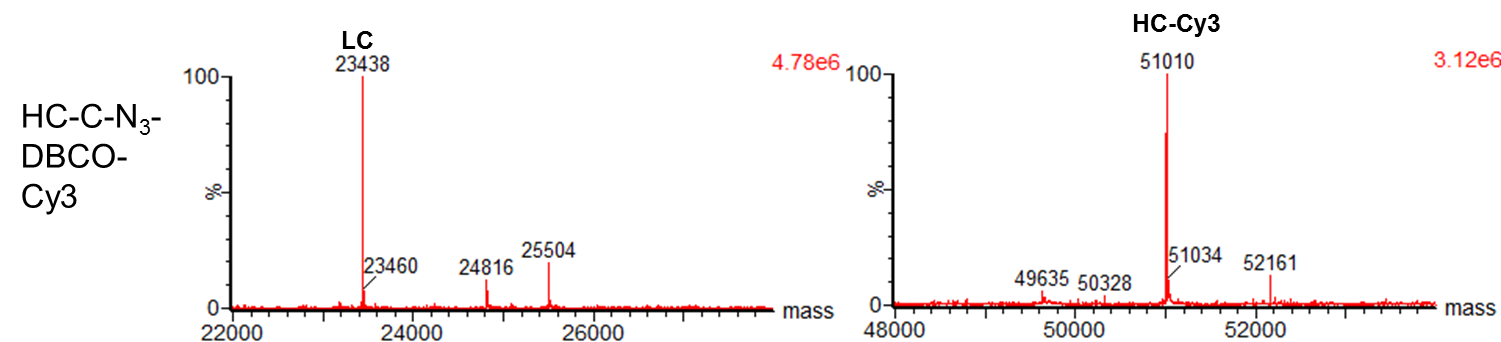
**

**Figure S12. Generating an antibody fluorophore conjugate using an improved SrtA variant.** LC/MS traces of a purified antibody-fluorophore conjugate generated using 5M/Y187L/E189R SrtA. The HC-C antibody was labelled with GGG-Lys(N3)-NH2 using 5M/Y187L/E189R and subsequently reacted with DBCO-Cy3. Light chain of the antibody remained unmodified.


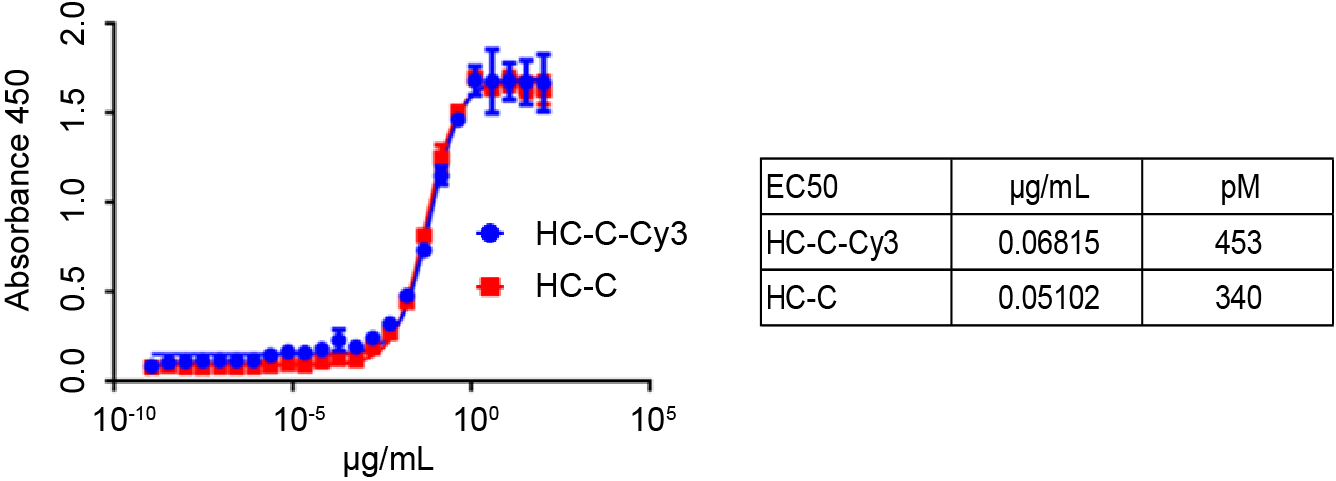


**Figure S13. Conjugation by Sortase did not affect antibody binding.** The HC-C anti-HER2 antibody was conjugated to the fluorescent dye Cy3 and binding to N87 HER2 expressing cells was measured for both the unmodified and conjugated antibody.


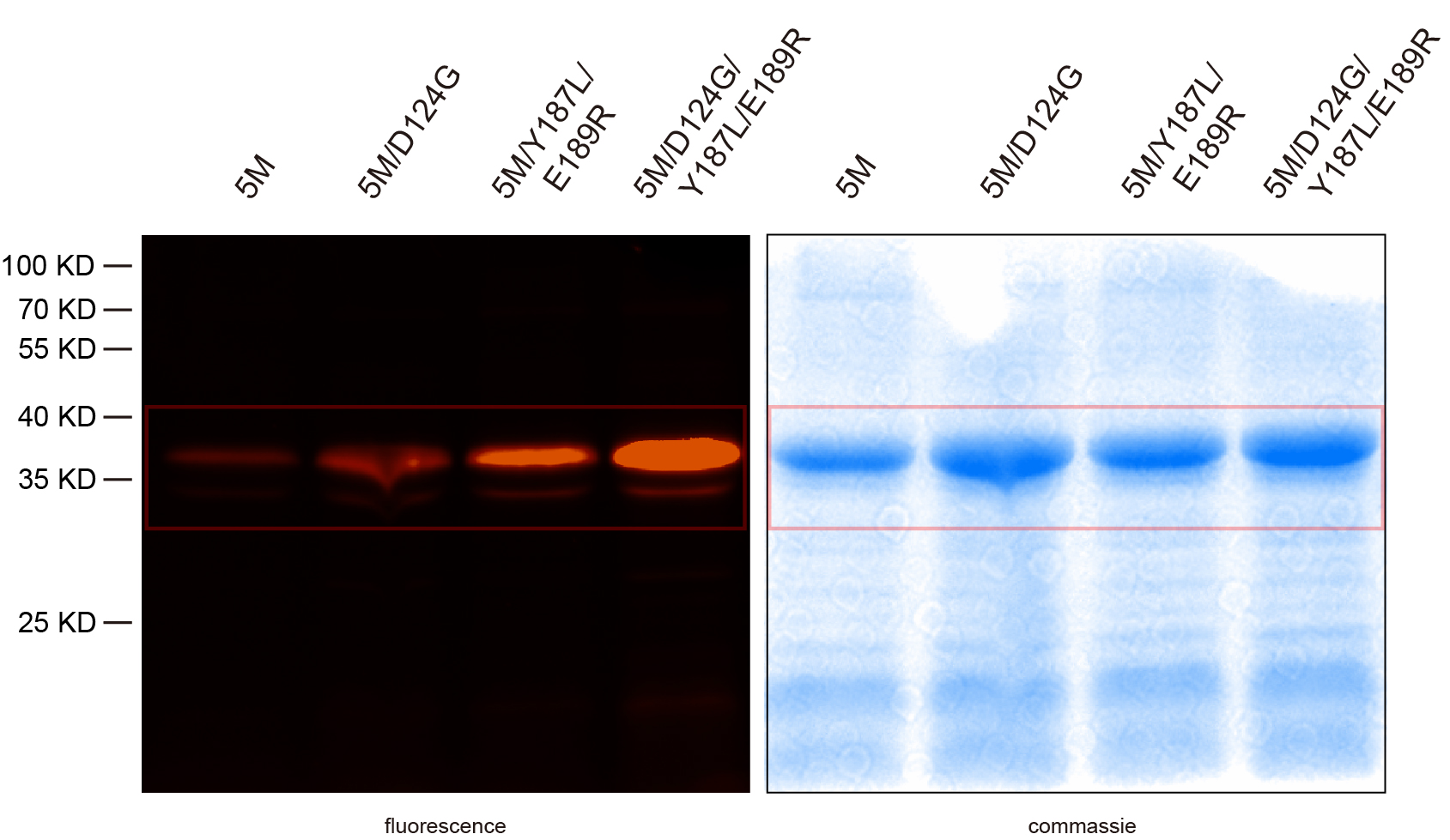


**Figure S14. Corresponding full images represented in Figure 3A.** Regions marked in red boxes represented the selected area in figure 3A.


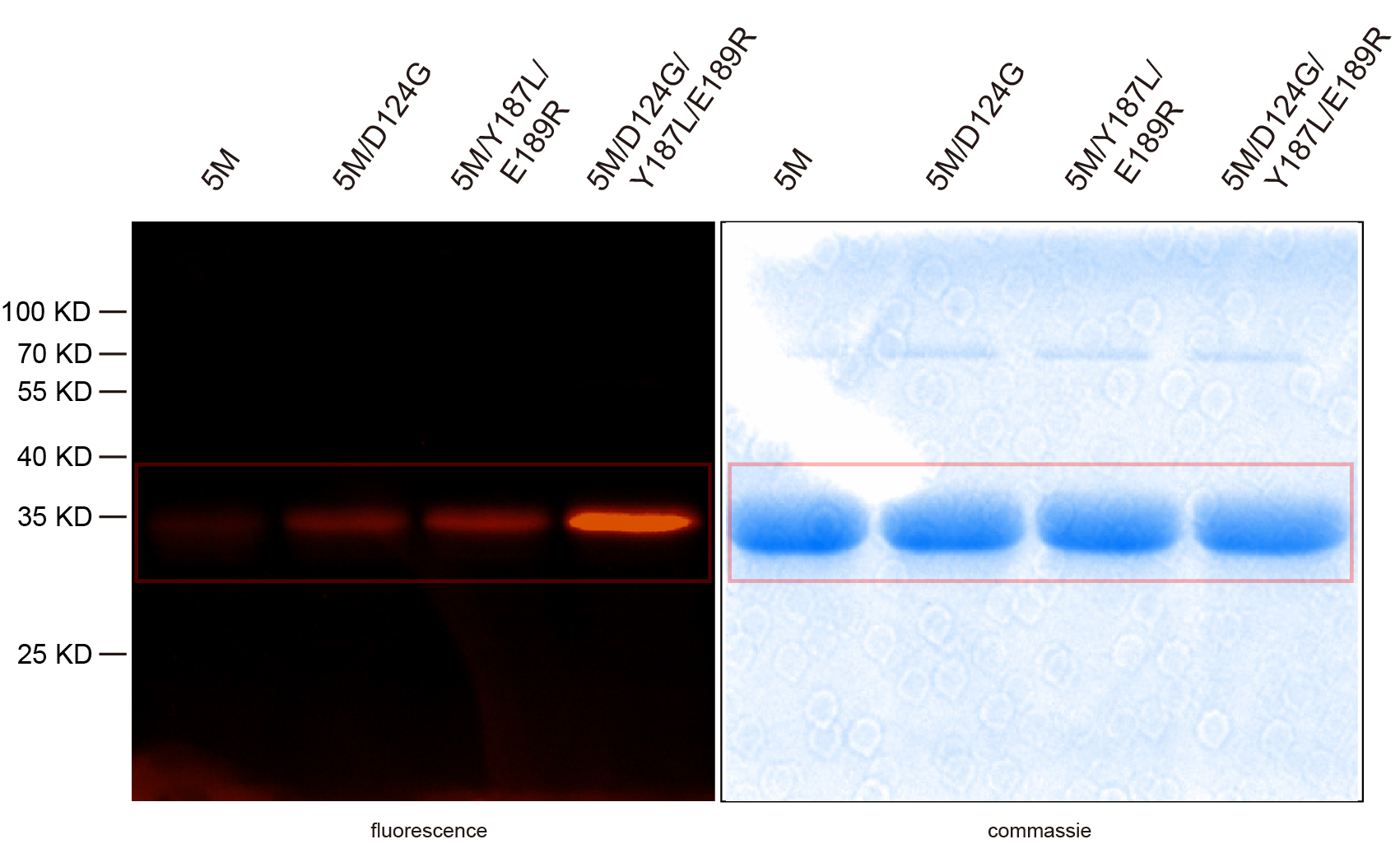


**Figure S15. Corresponding full gel images represented in Figure 3B.** Regions marked in red boxes represented the selected area in figure 3B.


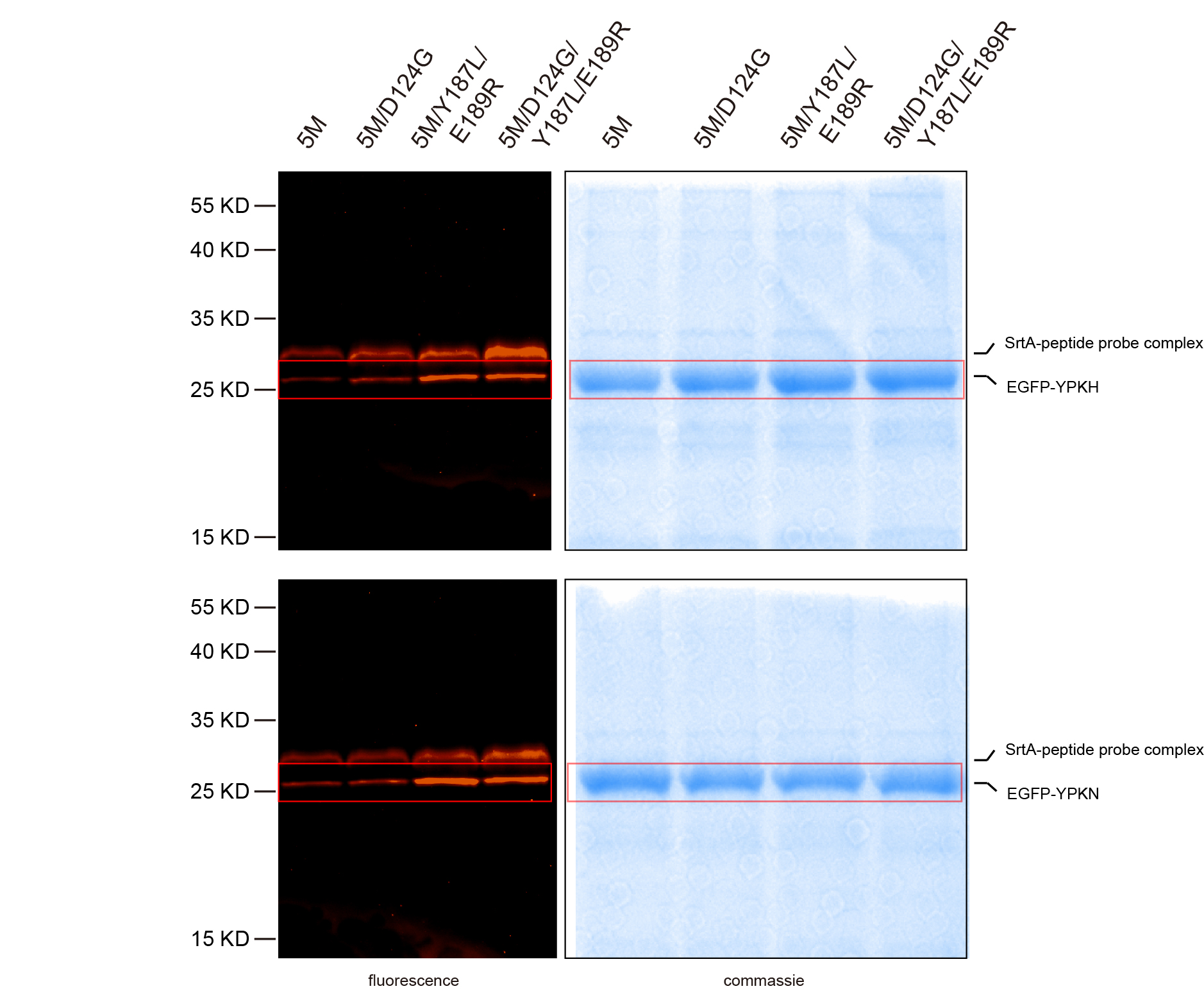


**Figure S16. Corresponding full gel images represented in Supplementary Figure 8.** Regions marked in red boxes represented the selected area. The SrtA bands could not be visualized in the commassie stained gel due to very low SrtA concentration (50 nM SrtA *vs.* 20 μM EGFP).

Protein sequences:

GGG-cpVenus:

GGGMHEEEFQFLRCQQCQAEAKCPKLLPCLHTLCSGCLEASGMQCPICQAPWPLGADTPALELMDGGVQLADHYQQNTPIGDGPVLLPDNHYLSYQSALSKDPNEKRDHMVLLEFVTAAGITLGMDELYKGGSGGMVSKGEELFTGVVPILVELDGDVNGHKFSVSGEGEGDATYGKLTLKLICTTGKLPVPWPTLVTTLGYGLQCFARYPDHMKQHDFFKSAMPEGYVQERTIFFKDDGNYKTRAEVKFEGDTLVNRIELKGIDFKEDGNILGHKLEYNYNSHNVYITADKQKNGIKANFKIRHNIELEHHHHHH

EGFP-LPETG:

MLNDIFEAQKIEWHEMVSKGEELFTGVVPILVELDGDVNGHKFSVSGEGEGDATYGKLTLKFICTTGKLPVPWPTLVTTLTYGVQCFSRYPDHMKQHDFFKSAMPEGYVQERTIFFKDDGNYKTRAEVKFEGDTLVNRIELKGIDFKEDGNILGHKLEYNYNSHNVYIMADKQKNGIKVNFKIRHNIEDGSVQLADHYQQNTPIGDGPVLLPDNHYLSTQSALSKDPNEKRDHMVLLEFVTAAGITLGMDELYKLPETGGLEHHHHHH

**Light Chain – C-Terminal-Sortag (LC-C)**

DIQMTQSPSSLSASVGDRVTITCRASQDVNTAVAWYQQKPGKAPKLLIYSASFLYSGVPSRFSGSRSGTDFTLTISSLQPEDFATYYCQQHYTTPPTFGQGTKVEIKRTVAAPSVFIFPPSDEQLKSGTASVVCLLNNFYPREAKVQWKVDNALQSGNSQESVTEQDSKDSTYSLSSTLTLSKADYEKHKVYACEVTHQGLSSPVTKSFNRGECGGLPETGGHHHHHH

**Light Chain – N-Terminal-Sortag (LC-N)**

GGGDIQMTQSPSSLSASVGDRVTITCRASQDVNTAVAWYQQKPGKAPKLLIYSASFLYSGVPSRFSGSRSGTDFTLTISSLQPEDFATYYCQQHYTTPPTFGQGTKVEIKRTVAAPSVFIFPPSDEQLKSGTASVVCLLNNFYPREAKVQWKVDNALQSGNSQESVTEQDSKDSTYSLSSTLTLSKADYEKHKVYACEVTHQGLSSPVTKSFNRGEC

**Heavy Chain C-Terminal-Sortag (HC-C)**

EVQLVESGGGLVQPGGSLRLSCAASGFNIKDTYIHWVRQAPGKGLEWVARIYPTNGYTRYADSVKGRFTISADTSKNTAYLQMNSLRAEDTAVYYCSRWGGDGFYAMDYWGQGTLVTVSSASTKGPSVFPLAPSSKSTSGGTAALGCLVKDYFPEPVTVSWNSGALTSGVHTFPAVLQSSGLYSLSSVVTVPSSSLGTQTYICNVNHKPSNTKVDKKVEPKSCDKTHTCPPCPAPELLGGPSVFLFPPKPKDTLMISRTPEVTCVVVDVSHEDPEVKFNWYVDGVEVHNAKTKPREEQYNSTYRVVSVLTVLHQDWLNGKEYKCKVSNKALPAPIEKTISKAKGQPREPQVYTLPPSREEMTKNQVSLTCLVKGFYPSDIAVEWESNGQPENNYKTTPPVLDSDGSFFLYSKLTVDKSRWQQGNVFSCSVMHEALHNHYTQKSLSLSPGGGLPETGGHHHHHH

**Heavy Chain N-Terminal-Sortag (HC-N)**

GGGEVQLVESGGGLVQPGGSLRLSCAASGFNIKDTYIHWVRQAPGKGLEWVARIYPTNGYTRYADSVKGRFTISADTSKNTAYLQMNSLRAEDTAVYYCSRWGGDGFYAMDYWGQGTLVTVSSASTKGPSVFPLAPSSKSTSGGTAALGCLVKDYFPEPVTVSWNSGALTSGVHTFPAVLQSSGLYSLSSVVTVPSSSLGTQTYICNVNHKPSNTKVDKKVEPKSCDKTHTCPPCPAPELLGGPSVFLFPPKPKDTLMISRTPEVTCVVVDVSHEDPEVKFNWYVDGVEVHNAKTKPREEQYNSTYRVVSVLTVLHQDWLNGKEYKCKVSNKALPAPIEKTISKAKGQPREPQVYTLPPSREEMTKNQVSLTCLVKGFYPSDIAVEWESNGQPENNYKTTPPVLDSDGSFFLYSKLTVDKSRWQQGNVFSCSVMHEALHNHYTQKSLSLSPG
